# Imputing longitudinal PROM data at the item and score level in clinical trials

**DOI:** 10.1186/s12874-026-02984-0

**Published:** 2026-09-04

**Authors:** Carlo A.G. Veltri, Ulrike Grittner, Pimrapat Gebert

**Affiliations:** 1 German Rheumatology Research Center, Berlin, 10117 Germany; 2 Institute of Biometry and Clinical Epidemiology, Charité – Universitätsmedizin Berlin, Berlin, 10117 Germany

**Keywords:** Missing data, Multiple imputation, Simulation study, Patient-reported outcome measures

## Abstract

**Background:**

Missing data in patient-reported outcome measures (PROMs) can affect estimation accuracy and inferential validity, particularly under missing-at-random (MAR) mechanisms. Multiple imputation (MI) is commonly recommended, but the relative performance of item-level and score-level imputation for longitudinal PROM composite scores remains insufficiently understood.

**Methods:**

We conducted a simulation study based on longitudinal clinical trial PROM data. Predictive mean matching (PMM) and random forest (RF) imputation were evaluated at both the item and score level under empirically calibrated MAR mechanisms. Simulation scenarios varied sample size, overall missingness, and the proportion of unit nonresponse. Performance was assessed using root mean squared error (RMSE), bias magnitude, relative efficiency compared with complete case analysis (CCA), conditional confidence interval coverage, and variance ratio estimates.

**Results:**

RMSE decreased with increasing sample size and increased with higher proportions of missingness across all methods. Both PMM and RF generally outperformed CCA in terms of RMSE and relative efficiency, particularly under higher missingness. Differences between item-level and score-level imputation were generally modest, although score-level imputation tended to yield slightly lower RMSE and bias magnitudes under more severe missingness conditions. Conditional coverage remained close to nominal levels across most MI settings. Variance tended to be overestimated in some scenarios, although sensitivity analyses with larger numbers of imputations substantially reduced this effect. PMM showed comparatively stable performance across simulation settings.

**Conclusions:**

Multiple imputation methods generally outperformed complete case analysis for handling longitudinal PROM data under MAR. While score-level imputation sometimes showed slightly more favorable performance than item-level imputation when targeting composite score means, the magnitude of these differences was frequently small in practical terms. PMM provided stable performance across a wide range of settings and represents a reasonable default approach for many applied PROM analyses.

**Supplementary Information:**

The online version contains supplementary material available at https://doi.org/10.1186/s12874-026-02984-0.

## Background

Missing data are a common source of bias in clinical studies, particularly when the probability of missingness depends on observed or unobserved variables related to the outcome. Ignoring such missingness can compromise the validity of study findings and, consequently, the clinical or regulatory decisions based on them, especially in evaluations of new drugs or therapeutic interventions. Multiple imputation (MI) is a widely accepted approach to mitigate bias by replacing missing values with multiple sets of plausible values that reflect uncertainty about the unobserved data. Despite extensive methodological development, the literature provides no clear consensus on which imputation strategies perform best under realistic data-generating mechanisms encountered in applied research. The present study contributes to this debate through a simulation-based evaluation of MI methods for patient-reported outcome measures (PROMs).

PROMs typically consist of standardized questionnaires designed to capture health outcomes directly from patients. They may be administered during the course of an illness or following an intervention to assess treatment impact. PROMs frequently target aspects of health-related quality of life, such as anxiety, pain, or social functioning, which represent latent constructs that cannot be observed directly. Validated questionnaires operationalize these constructs through multiple items, which are subsequently aggregated into quantitative scores suitable for statistical analysis.

In many applied analyses, inference focuses on the composite PROM score rather than on individual item responses. However, missing data may arise at both the item level (item nonresponse) and the questionnaire level (unit nonresponse), and MI can be applied at either level. This creates a methodological ambiguity between imputing items—thereby preserving the internal structure of the instrument—and imputing the derived score directly, which is often the estimand of interest. Under missing-at-random (MAR) mechanisms, item-level imputation may be expected to perform well when partially observed items are available as predictors, particularly in settings with sparse item nonresponse. Simulation evidence supporting this intuition has been reported [1].

Nevertheless, existing findings are somewhat inconsistent. Some studies report superior performance of item-level imputation at higher missingness rates [2], whereas others favor score-level imputation due to improved precision of estimates [3]. In contrast, Rombach et al. [4] find that the level of imputation has little impact in most scenarios, except in smaller samples (*n ≤ 200*), where score-level imputation appears preferable. Importantly, they also report convergence problems for item-level imputation in small samples, highlighting practical limitations that may outweigh theoretical advantages.

This simulation study aims to contribute to the body of research on multiple imputation of PROM data by providing a new data source for a realistic simulation of MAR data. We vary not only the overall proportion of missingness but also the relative proportion of unit nonresponse to investigate at which levels imputation at item or score level is preferable.

The simulation framework is informed by data from the Oncological Social Care Project of the BKK (OSCAR), an interventional, non-randomized palliative care study [5]. Missingness patterns are derived from the observed data and used to generate realistic MAR scenarios while varying the overall proportion of missing data, the contribution of unit nonresponse, and sample size. This design allows us to examine how imputation performance depends on both the level of imputation and the complexity of the data structure, thereby situating the comparison of methods in a setting that closely reflects applied analyses.

### Aims of this research

This simulation study aims to compare the performance of commonly used multiple imputation strategies for handling missing data in PROMs when missingness occurs both at the item level and as unit nonresponse. In particular, we contrast score-level imputation (imputing the composite score directly) with item-level imputation (imputing questionnaire items and then deriving the score) under an empirically calibrated missing-at-random mechanism motivated by clinical trial data.

The estimand throughout is the longitudinal marginal mean of the composite PROM score, i.e., the vector of marginal composite means at each time point. The simulation varies sample size, the overall proportion of incomplete observations, and the proportion of unit nonresponse among incomplete observations to identify settings in which item-level or score-level imputation is preferable in terms of accuracy.

In the remainder of the manuscript, method performance is summarized using standard simulation operating characteristics, including bias, root mean squared error (RMSE), conditional empirical coverage of the nominal 95% confidence interval relative to the complete-data reference, and calibration of variance estimates.

## Methods

### Data source and study context

This study is based on data from the OSCAR study, a non-randomized, multi-center intervention study investigating care provision for patients with advanced cancer [5]. OSCAR comprises three research modules: (i) patient-reported outcomes collected at baseline and during follow-up, (ii) health insurance claims data for cost-effectiveness analyses, and (iii) qualitative analyses of structured interviews. The present work exclusively uses data from the first module.

The OSCAR intervention introduces a palliative care program centered on Social Care Nurses (SCNs), who support patients by identifying gaps in healthcare utilization, facilitating access to medical and social services, and providing psychosocial support. The primary objective of the overall study is to improve quality of life in patients with advanced cancer through this patient-centered approach.

### Data and missingness characteristics

Patient-reported outcome measures (PROMs) were collected at baseline and at three follow-up visits over a one-year period. For the simulation study, the European Health Literacy Survey questionnaire short form (HLS-EU-Q6) was selected because it showed the highest proportion of missing values among the collected PROMs and was therefore particularly suitable for evaluating imputation strategies. Missingness rates among the other PROMs collected ranged from 18.3% up to 22.4%, as shown in the supplement. The HLS-EU-Q6 includes six items assessing perceived difficulties in accessing, understanding, appraising, and using health-related information. Responses are given on a four-point scale (“very difficult” to “very easy”), with “don’t know” treated as missing. As nonresponse might have a different missingness mechanism than “don’t know”, a sensitivity analysis with selected parameter constellations has been performed where “don’t know” has been assigned the interim value of 2.5. The score is calculated as the mean of the item responses when at least five items are answered, resulting in a score between 1 and 4, where higher values indicate higher perceived health literacy [6].

Among 1,210 observations, approximately 50% were complete. The most frequent missingness pattern was unit nonresponse (about 20%), followed by patterns with a single missing item (approximately 10% in total). Patterns with two or four missing items were rare (around 1.3% each). Imputation models were based solely on fully observed covariates that were preselected according to their presumed association with missingness, as suggested by the literature. These covariates included treatment group, study site, age, cancer diagnosis, and time point of data collection (Table 1).

**Table 1:** Covariates and descriptions

Covariate	Description
Group	Intervention vs. control
Study site	Recruiting study center
Age	Age in years
Cancer type	Dummy variables indicating diagnosis
Time point	Baseline and follow-up visits

### Simulation design and imputation methods

**Fig. 1:**
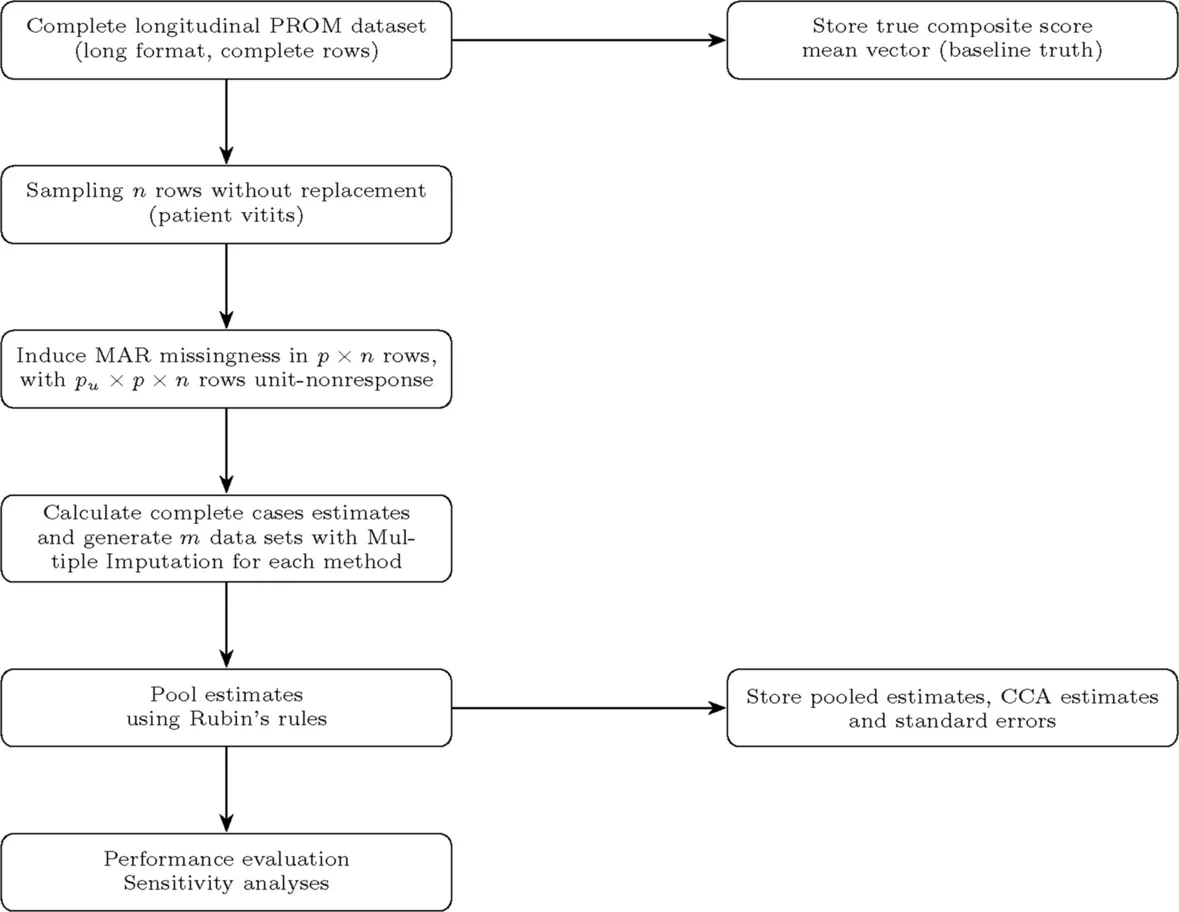
Simulation design and analysis workflow

Figure 1 shows the work flow for one thousand simulation runs for each parameter constellation of sample size, overall missingness, and thereof unit nonresponse. PROM observations were represented in long format with multiple rows per patient across time points. Each desired sample size was obtained by sampling without replacement at the row (person–time observation) level from the dataset consisting of completely observed rows (complete on all items). The sample size was drawn without replacement to preserve the covariate structure observed in the complete data set.

After sampling and saving the true value of the composite score mean vector one thousand times for each parameter constellation, a fraction of the data was set to missing according to the MAR-inducing algorithm described in the next subsection. The resulting dataset, with the desired overall proportion of incomplete rows and the desired proportion of unit nonresponse among incomplete rows, was then imputed using different MI approaches. For each method, the pooled estimate of the composite score mean vector and its Rubin’s rules standard error were stored.

To compare item- and score-level imputation strategies, two conceptually distinct approaches were selected: predictive mean matching (PMM) and random-forest (RF) imputation. PMM constitutes a widely recommended semi-parametric method within multiple imputation because it relaxes distributional assumptions and preserves observed data values under potential model misspecification [7]. Despite evidence indicating that PMM may exhibit bias under specific missing-at-random mechanisms [8], it remains a commonly used benchmark method in applied health and survey research and therefore provides an appropriate reference approach.

RF imputation represents a flexible non-parametric alternative that can accommodate nonlinear relationships, higher-order interactions, and mixed variable types frequently encountered in clinical and patient-reported outcome data. Previous investigations have reported robust performance across a range of missingness scenarios, particularly with increasing proportions of missing data [9]. However, RF-based imputation may introduce bias when the missingness mechanism depends strongly on the outcome variable [10].

The objective of this simulation study was not to determine a universally optimal imputation procedure but to assess whether conclusions regarding item- versus score-level imputation depend on the underlying modeling paradigm. The comparison between PMM and RF therefore contrasts a semi-parametric regression-based approach with a flexible machine-learning method representing differing assumptions about functional form and model specification. Fully parametric ordinal models, such as proportional-odds models, were not considered further because convergence difficulties have been reported in settings with small sample sizes and higher levels of missingness [4], conditions reflected in several simulation scenarios examined here.

Performance was assessed using root mean squared error (RMSE), bias, and relative efficiency compared to complete cases analysis (CCA), as well as conditional coverage and variance estimation accuracy. For the random forest approach, default hyperparameter settings as implemented in the respective R packages were used. Imputations were conducted using the R package mice [11] with R version 4.4 [12]. Longitudinal predictive mean matching was implemented via sampling at each time point with *d = 5* donors. For each simulated incomplete dataset, *m = 5* imputations were generated and pooled according to Rubin’s rules. Some arguments have been provided for increasing the number of imputations in practice [13, 14]. Due to the large number of parameter constellations tested, the number of imputations was kept lower in this simulation to maintain computational feasibility. A sensitivity analysis has been performed using *m= 20* imputations for selected parameter constellations.

### Simulation of MAR data

The missingness mechanism follows an algorithm by van Buuren et al. [15], which was outlined and adapted in several further publications [1, 4, 16] (Algorithm 1) and adapted to explicitly vary the proportion of unit-non response. The algorithm simulates MAR missingness by first randomly assigning each case to one of the missing data patterns occurring in the original data set. The relative frequency of unit-non response is explicitly set to the desired proportion, while the other frequencies are proportional to their actual occurrence in the data set. A linear score is assigned to each case based on the probability of belonging to their respective pattern. The probability is estimated by means of covariate-based logistic models predicting the occurrence of the respective pattern. Pattern-specific missingness probabilities are derived from these scores (via subgroup odds) to achieve the target proportion of incomplete cases, and missing values are imposed by comparing these probabilities to uniform random draws.

**Figure:**
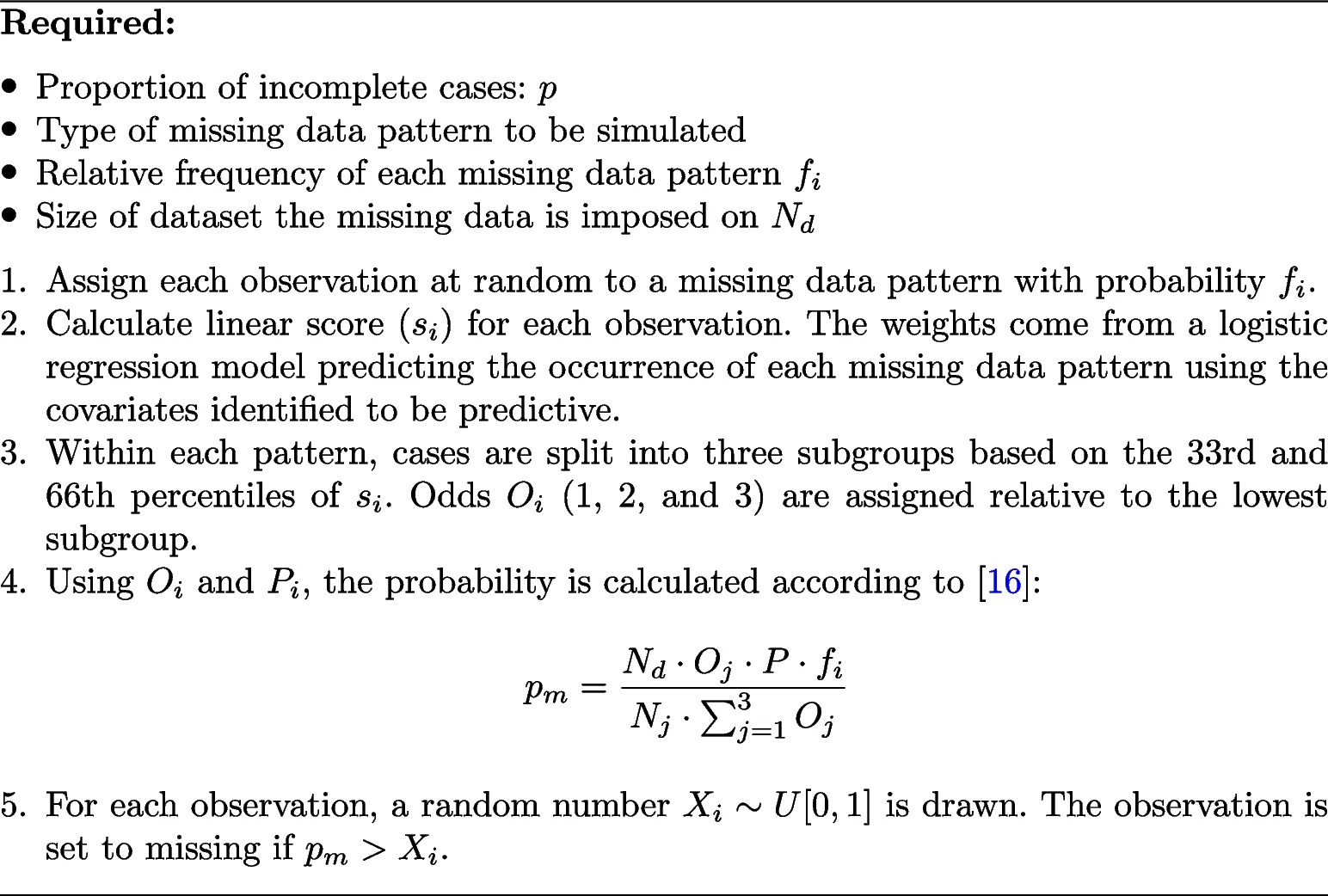
**Algorithm 1** Algorithm for imposing MAR data [15]

### Performance measures

As the estimand in this simulation study is the vector of mean composite scores, point estimation accuracy was evaluated using the root mean squared error (RMSE) of the composite score. The RMSE was defined as the euclidean norm of the differences of estimated and true mean composite scores at each time point. To allow comparison with complete case analysis (CCA), relative efficiency (RE) was calculated as the ratio of the MSE under CCA to the MSE of the respective imputation method. Values greater than 1 indicate improved efficiency compared to CCA, whereas values below 1 reflect inferior performance. Magnitude of bias was defined as the euclidean norm of the bias vector, i.e. the norm of the mean differences at each time point. Bias can hereby not be assessed in terms of direction. However, averaging over the bias vector would allow for biases in different time points to cancel out, while the euclidean norm preserves bias magnitude.

Since the true data generating process is unknown, coverage could not be assessed directly. We instead define conditional coverage as the number of simulations in which the pointwise 95% confidence interval (CI) of the imputed composite mean vector (or complete cases mean vector) contains the respective pointwise mean of the fixed complete cases data set. Conditional coverage is expected to reach 100% if the sample size approaches the size of the fixed complete cases data set. A conditional coverage below 95% indicates anticonservative (i.e. too narrow) confidence intervals.

The variance ratio was defined as the ratio between the mean estimated variance of the mean vector within a replication divided by the observed (realized) variance of the estimated mean vector across simulations. A variance ratio of 1 indicates that the estimated variance matches the empirically observed variance, whereas a variance ratio above 1 indicates that the estimated variance after imputation is too large, meaning that the uncertainty was overestimated.

Table 2 shows the parameters that were investigated for this simulation study. Throughout this simulation, both the proportion of incomplete cases *p* as well as the relative frequency *f* of unit nonresponses have been varied. For higher frequencies of unit-nonresponse, score level imputation is assumed to perform relatively better compared to higher proportions of item-nonresponse. The relative frequencies among all item-nonresponse patterns were kept constant in this study.

**Table 2:** Simulation design factors and their levels

Factor	Levels
Imputation method	Predictive Mean Matching (PMM)
	Random Forest Imputation (RF)
	Complete Cases Analysis (CCA)
Sample size	50,100,200, 300, 400, 500
Proportion missing cases	0.1, 0.2, 0.3,0.5
Proportion unit-nonresponse	0.1, 0.2, 0.3,0.5

## Results

All models converged successfully in each simulation run.

**Fig. 2:**
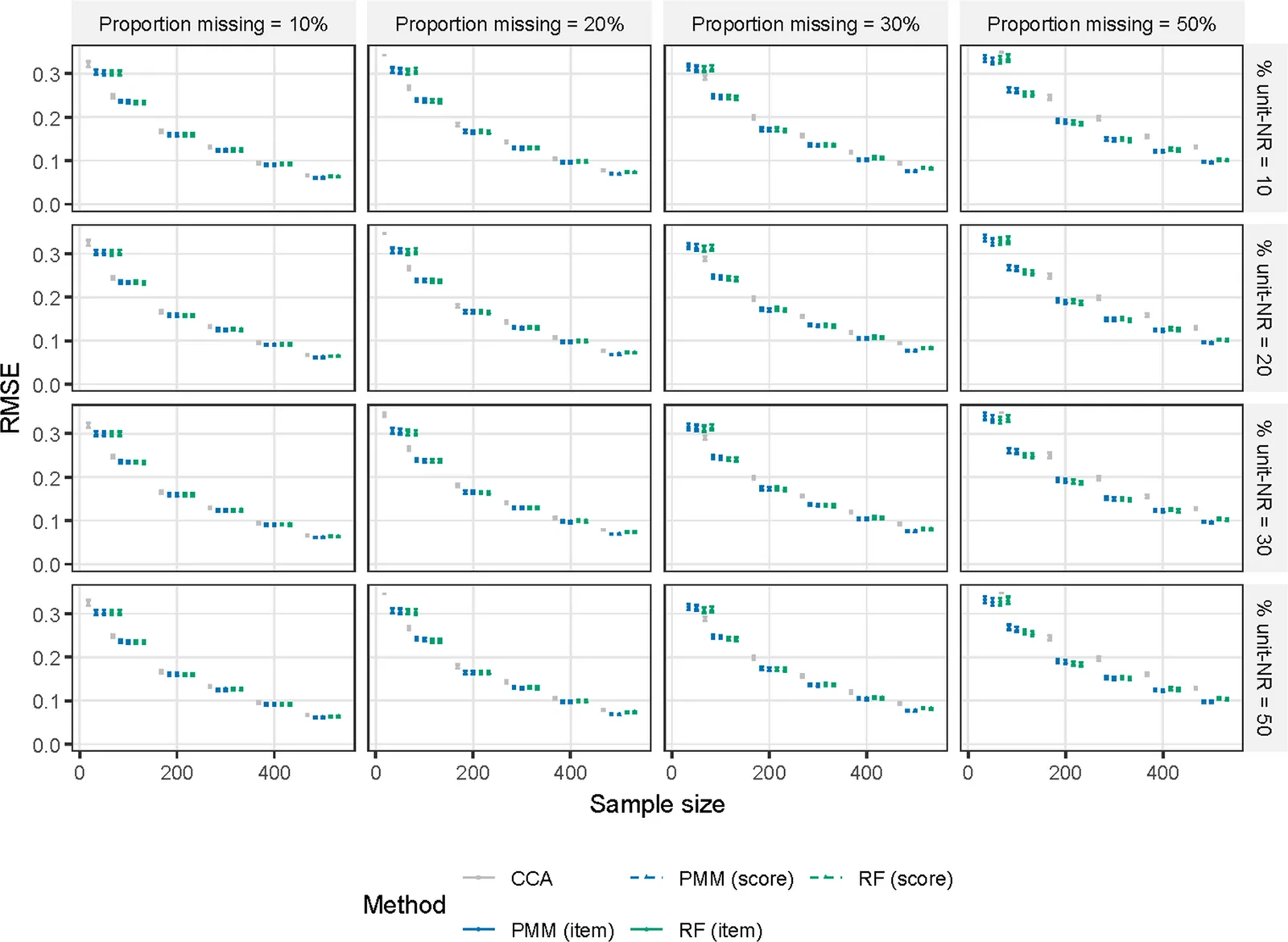
RMSE estimates for all parameter constellations along with 95%-confidence intervals. Abbreviations: RMSE, root mean squared error; % unit-NR, proportion of unit nonresponse; PMM, predictive mean matching; RF, random forest imputation; CCA, complete case analysis

Figure 2 presents RMSE estimates across all simulation conditions and imputation methods (Numerical results corresponding to all plots are provided in the Supplementary Material). RMSE decreased substantially with increasing sample size across all methods. For *n = 50*, RMSE values were approximately 30%, decreasing to around 8% under 10% MAR for *n = 400*. Under higher missingness (*50%* MAR), RMSE remained elevated at approximately 10% even for larger sample sizes. The largest reduction in RMSE was observed between *n = 50* and *n = 200*, where decreases of up to 18 percentage points occurred.

**Fig. 3:**
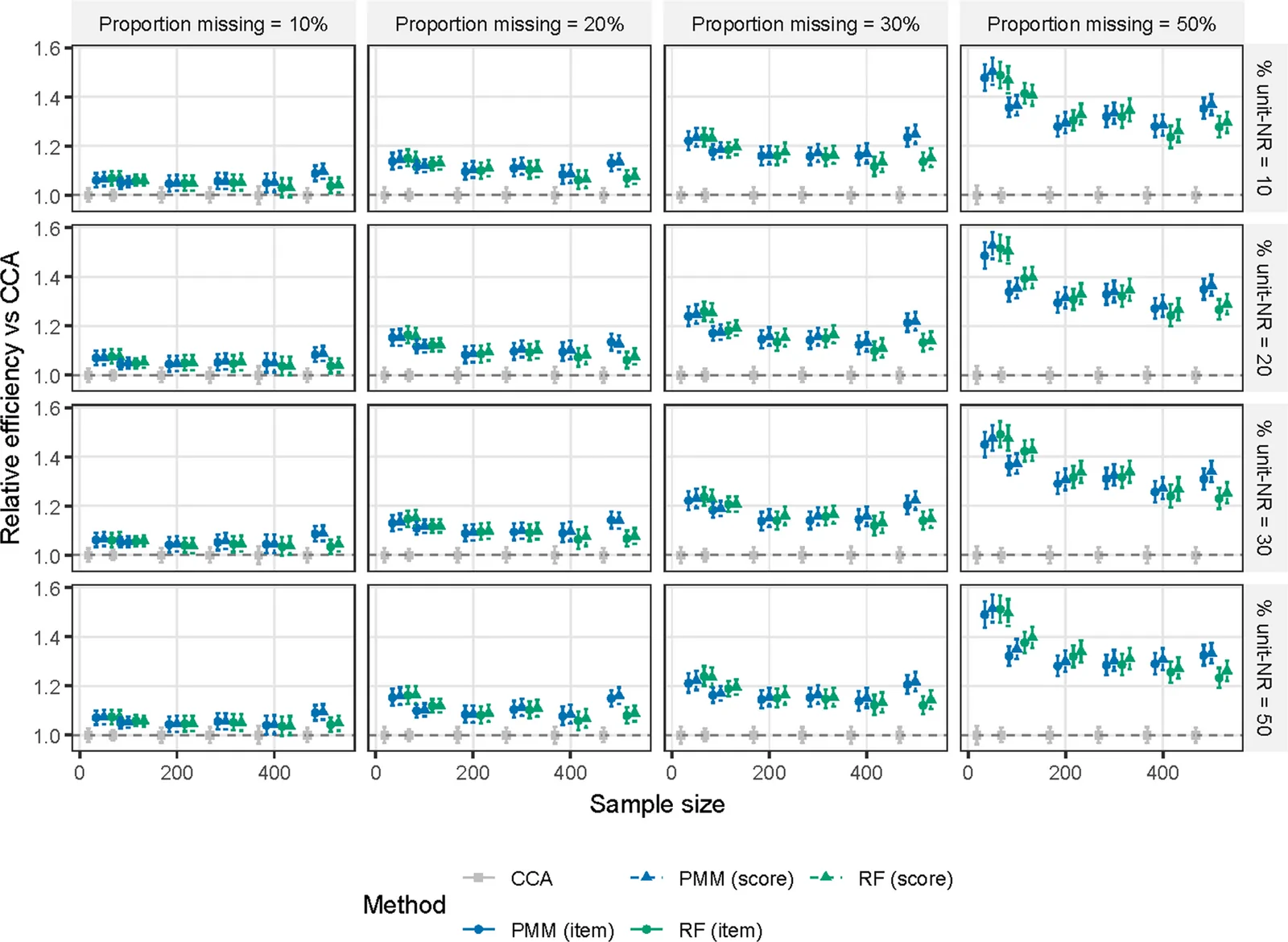
Relative efficiency estimates for all parameter constellations along with 95%-confidence intervals. Abbreviations: RMSE, root mean squared error; % unit-NR, proportion of unit nonresponse; PMM, predictive mean matching; RF, random forest imputation; CCA, complete case analysis

Across all parameter constellations, multiple imputation methods outperformed complete case analysis in terms of RMSE. Differences between MI and CCA became more pronounced with increasing proportions of MAR data and increasing proportions of unit nonresponse among MAR observations. Relative efficiency estimates shown in Fig. 3 further illustrate these differences. For lower missingness levels (*10%* MAR), relative efficiency of MI methods was typically around 5% higher than CCA, whereas under *50%* MAR, efficiency gains increased to approximately 50%. PMM and RF generally performed very similarly, although RF showed a slight tendency toward improved performance at higher proportions of unit nonresponse. Within methods, score-level imputation tended to perform marginally better than item-level imputation under higher proportions of unit nonresponse and overall missingness, although these differences were generally small.

**Fig. 4:**
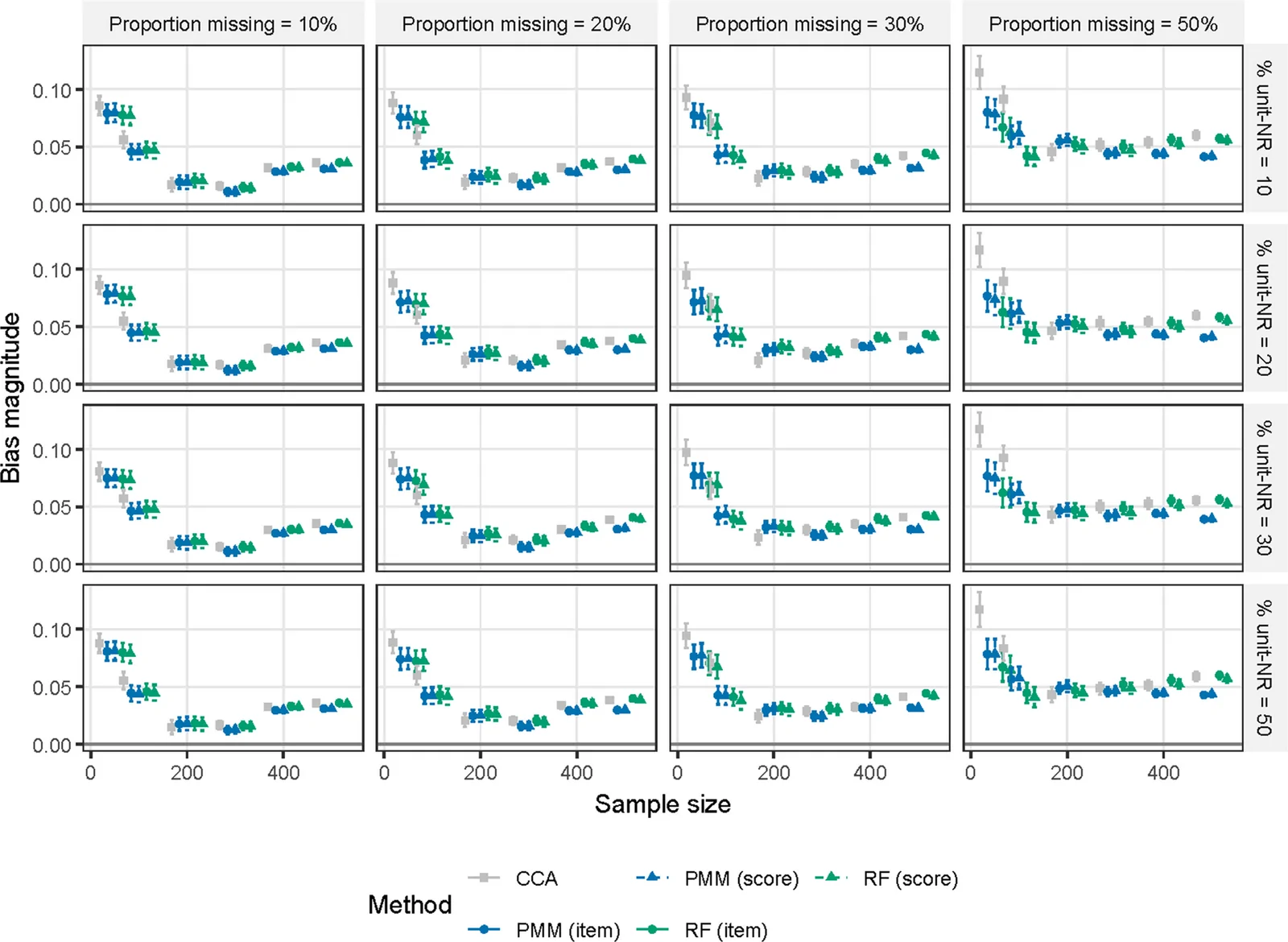
Bias magnitude estimates for all parameter constellations along with 95%-confidence intervals. Abbreviations: RMSE, root mean squared error; % unit-NR, proportion of unit nonresponse; PMM, predictive mean matching; RF, random forest imputation; CCA, complete case analysis

Bias magnitude estimates are shown in Fig. 4. In contrast to RMSE, bias showed only limited dependence on sample size beyond small samples. Higher bias magnitudes were primarily observed for *n = 50* and *n = 100*, whereas estimates remained comparatively stable for larger samples. For MAR proportions up to 30%, bias magnitude was generally around 0.08–0.10, corresponding to approximately 3–4% of overall mean true value (2.6). Under *50%* MAR, bias increased to approximately 0.13, corresponding to around 5% of the true value. MI and CCA yielded comparable bias magnitudes in larger samples, although CCA produced more biased estimates in smaller samples, with bias magnitudes reaching approximately 0.26 (10% of the true value) and up to approximately 0.31 (12% of the true value) for *n = 50* and *50%* MAR. Differences between PMM and RF, as well as between item- and score-level imputation, were generally small, although score-level imputation tended to yield slightly lower bias under higher missingness conditions.

**Fig. 5:**
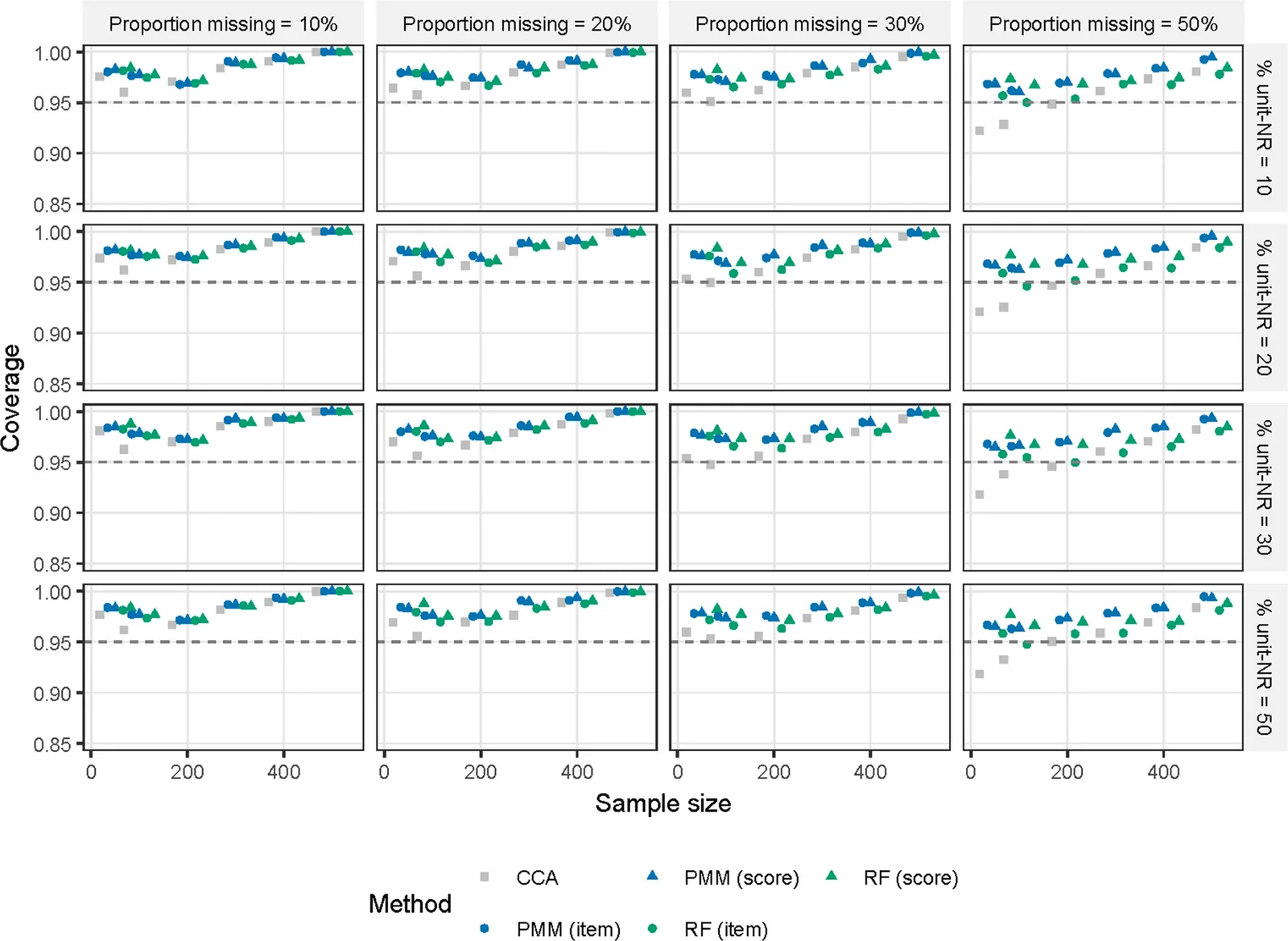
Conditional coverage estimates for all parameter constellations. Abbreviations: RMSE, root mean squared error; % unit-NR, proportion of unit nonresponse; PMM, predictive mean matching; RF, random forest imputation; CCA, complete case analysis

Figure 5 displays conditional coverage estimates across all simulation settings (Conditional coverage was defined as the proportion of confidence intervals from the incomplete-data analyses that contained the corresponding true parameter value estimated from the complete-case data set. Coverage should therefore ideally range between 95% and 100%). This criterion was met across nearly all parameter constellations. For MI methods, conditional coverage remained above 95% throughout all investigated settings and approached 100% for MAR proportions up to 30% in larger samples (*n ≥ 500*). Only CCA under severe missingness conditions (*50%* MAR and *n ≤ 100*) failed to attain the nominal coverage level, with conditional coverage decreasing to approximately 92%.

**Fig. 6:**
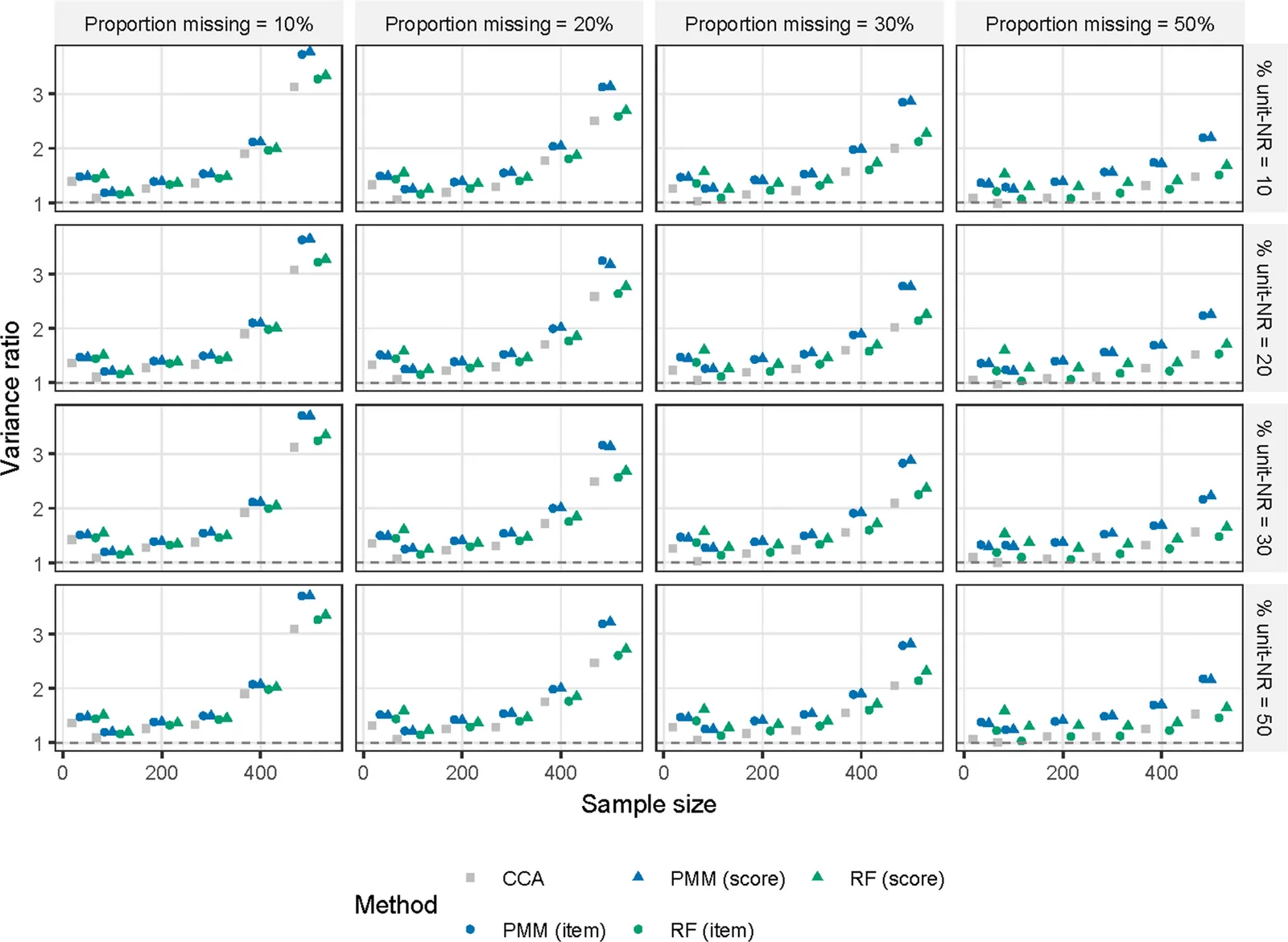
Variance ratio estimates for all parameter constellations. Abbreviations: RMSE, root mean squared error; % unit-NR, proportion of unit nonresponse; PMM, predictive mean matching; RF, random forest imputation; CCA, complete case analysis

Variance ratio estimates are shown in Fig. 6. Across most settings with moderate to high missingness, variance ratios ranged between 1 and 2, indicating moderate overestimation of variance. In larger samples (*n ≥ 400*), variance ratios increased up to approximately 3 for 20% MAR and even higher under 10% MAR. CCA generally yielded slightly lower variance overestimation compared to MI methods. Overall, the results indicate that MI methods improved estimation accuracy and coverage performance relative to CCA, while at the same time tending to overestimate variance to a greater extent in larger samples.

Sensitivity analyses were conducted for sample sizes *n = 200* and *n = 400* under 20% and 50% MAR, combined with 20% and 50% unit nonresponse among MAR observations. Simulations were repeated using (1) *m = 20* imputations and (2) coding don’t know” responses as an interim value of 2.5. Corresponding plots and numerical tables are provided in the Supplementary Material. Increasing the number of imputations to *m = 20* resulted in RMSE and bias estimates comparable to the main analyses. However, variance ratio estimates decreased substantially from approximately 2 to values between 1.0 and 1.2, indicating more accurate variance estimation. Treating don’t know” responses as 2.5 likewise produced similar RMSE and bias estimates, while reducing variance ratios to approximately 1.4. Conditional coverage decreased slightly under this specification, reaching approximately 95% for 50% MAR and 97% for 20% MAR.

## Discussion

Under empirically calibrated MAR mechanisms derived from PROM data, both the imputation method and the level of imputation (item vs. score) influenced estimation performance of the composite score mean, although the magnitude of these differences varied across performance measures and simulation settings. RMSE decreased substantially with increasing sample size, while bias magnitudes were primarily driven by the extent of missingness rather than sample size. Higher proportions of overall missingness and unit nonresponse were consistently associated with increased RMSE and bias across all methods.

Compared with complete case analysis (CCA), multiple imputation (MI) methods generally achieved lower RMSE and higher relative efficiency, particularly under larger proportions of missing data. However, absolute differences between methods were often modest in practical terms, especially under low to moderate missingness. Relative efficiency gains became more pronounced only under more severe missingness conditions, where MI approaches retained substantially more information than CCA.

Differences between item-level and score-level imputation were likewise generally small. Score-level imputation tended to yield slightly lower RMSE and bias magnitudes under higher proportions of missingness and unit nonresponse. These advantages were more visible for random forest imputation, whereas predictive mean matching (PMM) showed comparatively similar performance across imputation levels. At the same time, the observed differences were typically considerably smaller than the differences attributable to the overall proportion of missing data itself. Consequently, the practical relevance of the imputation level may depend strongly on the degree of missingness and the precision requirements of the intended analysis.

The additional performance measures further contextualize these findings. Conditional coverage remained close to or above the nominal 95% level across nearly all MI settings and approached 100% in larger samples with lower missingness. In contrast, CCA failed to attain nominal coverage only under the most extreme missingness conditions. These findings suggest that MI approaches were generally successful in preserving inferential validity under the investigated MAR mechanisms.

Variance ratio estimates indicated moderate overestimation of variance across methods, particularly in larger samples and lower MAR settings. While CCA tended to yield slightly lower variance overestimation than MI methods, sensitivity analyses demonstrated that this pattern was strongly influenced by the number of imputations. Increasing the number of imputations from *m = 5* to *m = 20* substantially improved variance estimation while leaving RMSE and bias largely unchanged, suggesting that part of the observed variance inflation reflected Monte Carlo variability rather than systematic deficiencies of the imputation models themselves.

Overall, the findings support previous evidence that MI approaches leveraging observed predictors are preferable to CCA under MAR in PROM settings (cf. [17, 18]). At the same time, the results suggest that the practical importance of selecting between item- and score-level imputation may often be limited in settings with low or moderate missingness, where all investigated MI approaches performed similarly. The primary practical implication may therefore be less the identification of a universally superior imputation strategy and more the demonstration that avoiding CCA becomes increasingly important as missingness rises.

### Limitations

This study contributes to simulation-based research on missing data in PROMs by using empirically calibrated MAR mechanisms reflecting realistic clinical settings. Nevertheless, the investigated parameter constellations necessarily constrain the scope of inference, and the findings should therefore be interpreted as context-specific rather than universally generalizable.

The simulation relied on MAR mechanisms informed by covariates previously associated with item nonresponse (e.g., [17, 18]). The applicability of the results therefore depends on whether similar predictors are available and adequately capture the missingness process in applied settings. If important determinants of missingness remain unobserved, missing-not-at-random (MNAR) mechanisms may arise, potentially altering both overall method performance and the relative differences between item- and score-level imputation.

The estimand considered in this study was the vector of mean composite scores across longitudinal measurements. Consequently, the findings are directly relevant to analyses targeting average longitudinal PROM trajectories at the population level. However, they may not generalize to other longitudinal estimands, such as subject-specific trajectories, random effects, transition parameters, or time-to-event outcomes derived from PROM data. In particular, the simulation focused on estimation accuracy of marginal mean structures rather than preservation of the full covariance structure or within-subject dependence. Different conclusions may therefore emerge in settings where individual longitudinal dynamics or covariance estimation are of primary interest.

Interpretation of the variance ratio findings also warrants caution. The observed variance overestimation was substantially reduced in sensitivity analyses using larger numbers of imputations, indicating that some results may reflect finite-imputation effects rather than structural shortcomings of the imputation procedures. Similarly, treating “don’t know” responses as an intermediate response category affected variance estimation and coverage more strongly than RMSE or bias, illustrating that apparently minor preprocessing decisions may influence inferential properties.

Finally, computational constraints limited the number of simulation runs per parameter constellation to one thousand. While sufficient to identify broad trends and stable qualitative patterns, additional simulation runs could provide more precise estimates and further clarify small differences between imputation strategies in extreme parameter settings.

## Conclusion

### Main findings

This simulation study evaluated multiple imputation techniques for handling missing PROM data under empirically calibrated MAR mechanisms, with a particular focus on comparing item-level and score-level imputation. Across most investigated settings, MI approaches achieved lower RMSE and higher relative efficiency than CCA, particularly under higher proportions of missingness.

Differences between item- and score-level imputation were generally modest in absolute magnitude, with score-level imputation sometimes yielding slightly lower RMSE and bias magnitudes. PMM demonstrated comparatively stable performance across parameter constellations, whereas RF showed somewhat greater sensitivity to the level of imputation. However, the practical magnitude of these differences was small relative to the overall effects of increasing missingness.

The additional analyses of conditional coverage and variance ratios further suggest that MI approaches generally preserved inferential validity under the investigated MAR mechanisms. Although variance tended to be overestimated in some scenarios, sensitivity analyses suggested that this effect was substantially reduced when larger numbers of imputations were used.

### Practical implications

For applied analyses of missing PROM data, the findings suggest several practical considerations rather than strict methodological rules.

First, when the target estimand is a composite score mean, score-level imputation appears to provide performance that is at least comparable to—and in some scenarios slightly better than—item-level imputation. However, the magnitude of these differences was frequently small. Consequently, the practical benefit of score-level imputation should be weighed against considerations such as model interpretability, computational complexity, and compatibility with the substantive research question.

Second, PMM may represent a reasonable default choice for many PROM applications due to its comparatively stable performance across simulation settings. Nevertheless, differences between PMM and RF were often modest, and both MI approaches generally outperformed CCA under increasing missingness.

Third, the findings emphasize that the proportion and structure of missing data are likely more practically consequential than the specific choice between item- and score-level imputation. Under severe missingness, both RMSE and bias increased across all methods, while efficiency losses associated with CCA became increasingly pronounced.

Finally, the sensitivity analyses indicate that implementation details such as the number of imputations and the treatment of ambiguous response categories may meaningfully influence inferential properties, particularly variance estimation and coverage. Applied analyses should therefore consider these modeling decisions explicitly rather than treating them as purely technical choices.

### Contribution

By extending previous work using empirically calibrated missingness mechanisms and a broader range of simulation settings, this study contributes additional evidence regarding the performance of MI approaches for PROM data under MAR. Rather than identifying a single universally optimal method, the findings help contextualize the magnitude and practical relevance of differences between commonly used imputation strategies. In particular, the results highlight that while score-level imputation and PMM often performed favorably, the largest performance differences were associated with the overall extent of missingness and the decision to avoid CCA under substantial incomplete data.

## Supplementary Information

Supplementary Material 1.

## Data Availability

The simulation was based on clinical trial data that are protected for patient confidentiality and cannot be publicly shared.

## Abbreviations

PROM: Patient-reported Outcome Measures
OSCAR: Oncological Social Care Project of the BKK
RMSE: Root mean squared error
MAR: Missing-at-random
MSE: Mean squared error
CCA: Complete cases analysis
PMM: Predictive Mean Matching
RF: Random Forest
NR: Non-response
MNAR: Missing-not-at-random
