# Supplementary material for: Imputing longitudinal PROM data at the item and score level in clinical trials
Source: BMC Med Res Methodol. 2026 Sep 4;26:210. doi: 10.1186/s12874-026-02984-0 (PMC13621587; doi:10.1186/s12874-026-02984-0)
Supplement: Supplementary file 1 [file 12874_2026_2984_MOESM1_ESM.zip › Supplementary.pdf]

1 Supplementary information

1.1 Dependency between covariates and items

Covariate	Item 1	Item 2	Item 3	Item 4	Item 5	Item 6
Age	0.047	0.049	−0.006	−0.033	0.011	−0.078
Diagnosis	0.112	0.082	0.099	0.107	0.107	0.114
Group	0.116	0.027	0.055	0.050	0.025	0.051
Site	0.164	0.163	0.136	0.170	0.151	0.149
Time	−0.030	−0.011	−0.018	−0.030	−0.055	−0.011

Categorical covariates (group, site, diagnosis) were analyzed using Cramer's V based on Pearson's chi-square tests. Numeric covariates (age and time) were analyzed using Spearman rank correlations. All analyses were conducted pairwise between each covariate and each item using complete cases only.

1.2 Observed missingness patterns

Full list of observed missingness patterns in the original dataset.

Table 1: Observed missingness patterns

Pattern	Frequency
111111	601
000000	235
011111	40
110111	39
111101	25
111110	24
110101	21
110000	16
111010	16
111011	16
101111	14
111000	11
010000	8
010111	8
011110	8
110011	8
110110	8
111100	8
000111	6
001111	6
110010	6
110100	6
011011	5
100111	5
110001	5
111001	5
010010	4
010011	4
010101	4
011100	4
011101	4
001100	3
010001	3
011010	3
101110	3
000100	2
001010	2
001011	2
001101	2
010110	2
011000	2
011001	2
100000	2
100110	2
101011	2
000001	1
001110	1
100011	1
100100	1
100101	1
101010	1
101100	1
101101	1

1.3 Predictors of missingness

Logistic regression output of covariates predicting the presence of any missing data.

Table 2: Logistic regression model predicting having any missingness

Covariate	Odds ratio	2.5% CI	97.5% CI
Group: Control	0.951	0.741	1.221
Site: D	1.692	1.233	2.327
Site: L	0.657	0.474	0.907
Age	1.021	1.011	1.031
Diagnosis: Lymphoma	1.247	0.851	1.828
Diagnosis: Malignant neoplasm of bronchus and lung	1.641	1.076	2.514
Diagnosis: Metastasized colorectal cancer	1.131	0.771	1.660
Diagnosis: Malignant neoplasm of pancreas	1.584	0.922	2.735
Diagnosis: Multiple myeloma and malignant plasma cell neoplasms	0.854	0.514	1.412
Diagnosis: Metastasized malignant neoplasm of breast	2.049	0.941	4.574
Diagnosis: Others	1.548	0.933	2.581
Time	0.997	0.970	1.024

1.4 Pattern-specific missingness models

In the missingness patterns, 0 indicates not missing and 1 indicates missing.

Table 3: Logistic missingness model for pattern 000000

Covariate	Odds ratio	2.5% CI	97.5% CI
Time: 3	0.860	0.632	1.170
Time: 6	1.019	0.737	1.409
Time: 12	0.994	0.711	1.390
Site: D	0.596	0.434	0.816
Site: L	1.523	1.103	2.109
Age	0.979	0.970	0.989
Diagnosis: Lymphoma	0.800	0.545	1.172
Diagnosis: Malignant neoplasm of bronchus and lung	0.605	0.395	0.921
Diagnosis: Metastasized colorectal cancer	0.879	0.600	1.288
Diagnosis: Malignant neoplasm of pancreas	0.627	0.363	1.077
Diagnosis: Multiple myeloma and malignant plasma cell neoplasms	1.174	0.710	1.951
Diagnosis: Metastasized malignant neoplasm of breast	0.486	0.218	1.058
Diagnosis: Others	0.637	0.383	1.052

Note. Estimates shown as – indicate unstable estimates caused by complete or quasi-complete separation.

Table 4: Logistic missingness model for pattern 111111

Covariate	Odds ratio	2.5% CI	97.5% CI
Time: 3	3.427	2.193	5.470
Time: 6	3.372	2.116	5.473
Time: 12	4.523	2.843	7.341
Site: D	1.525	1.019	2.285
Site: L	1.284	0.847	1.956
Age	1.014	1.001	1.027
Diagnosis: Lymphoma	1.126	0.678	1.871
Diagnosis: Malignant neoplasm of bronchus and lung	2.032	1.227	3.393
Diagnosis: Metastasized colorectal cancer	0.930	0.562	1.543
Diagnosis: Malignant neoplasm of pancreas	1.782	0.921	3.407
Diagnosis: Multiple myeloma and malignant plasma cell neoplasms	0.664	0.296	1.373
Diagnosis: Metastasized malignant neoplasm of breast	1.154	0.397	2.946
Diagnosis: Others	1.006	0.512	1.914

Note. Estimates shown as – indicate unstable estimates caused by complete or quasi-complete separation.

Table 5: Logistic missingness model for pattern 100000

Covariate	Odds ratio	2.5% CI	97.5% CI
Time: 3	0.617	0.279	1.302
Time: 6	0.393	0.141	0.948
Time: 12	0.294	0.084	0.796
Site: D	0.898	0.398	1.984
Site: L	0.454	0.174	1.140
Age	1.010	0.984	1.038
Diagnosis: Lymphoma	0.863	0.300	2.385
Diagnosis: Malignant neoplasm of bronchus and lung	0.659	0.167	2.223
Diagnosis: Metastasized colorectal cancer	1.247	0.466	3.392
Diagnosis: Malignant neoplasm of pancreas	1.685	0.406	6.036
Diagnosis: Multiple myeloma and malignant plasma cell neoplasms	0.805	0.174	2.818
Diagnosis: Metastasized malignant neoplasm of breast	–	–	–
Diagnosis: Others	0.963	0.203	3.480

Note. Estimates shown as – indicate unstable estimates caused by complete or quasi-complete separation.

Table 6: Logistic missingness model for pattern 001000

Covariate	Odds ratio	2.5% CI	97.5% CI
Time: 3	1.627	0.688	4.017
Time: 6	1.293	0.494	3.381
Time: 12	1.291	0.474	3.452
Site: D	0.702	0.303	1.551
Site: L	0.425	0.163	1.045
Age	1.027	1.000	1.057
Diagnosis: Lymphoma	1.053	0.420	2.635
Diagnosis: Malignant neoplasm of bronchus and lung	0.902	0.283	2.678
Diagnosis: Metastasized colorectal cancer	0.323	0.070	1.122
Diagnosis: Malignant neoplasm of pancreas	0.759	0.109	3.279
Diagnosis: Multiple myeloma and malignant plasma cell neoplasms	0.221	0.012	1.188
Diagnosis: Metastasized malignant neoplasm of breast	1.950	0.282	8.324
Diagnosis: Others	1.543	0.452	4.707

Note. Estimates shown as – indicate unstable estimates caused by complete or quasi-complete separation.

Table 7: Logistic missingness model for pattern 000010

Covariate	Odds ratio	2.5% CI	97.5% CI
Time: 3	0.580	0.197	1.549
Time: 6	0.458	0.125	1.361
Time: 12	0.506	0.138	1.509
Site: D	1.206	0.423	3.340
Site: L	0.739	0.223	2.370
Age	0.989	0.959	1.021
Diagnosis: Lymphoma	2.253	0.657	8.884
Diagnosis: Malignant neoplasm of bronchus and lung	1.152	0.208	5.832
Diagnosis: Metastasized colorectal cancer	1.943	0.533	8.082
Diagnosis: Malignant neoplasm of pancreas	1.880	0.234	11.274
Diagnosis: Multiple myeloma and malignant plasma cell neoplasms	1.469	0.197	7.957
Diagnosis: Metastasized malignant neoplasm of breast	–	–	–
Diagnosis: Others	–	–	–

Note. Estimates shown as – indicate unstable estimates caused by complete or quasi-complete separation.

Table 8: Logistic missingness model for pattern 000001

Covariate	Odds ratio	2.5% CI	97.5% CI
Time: 3	0.473	0.127	1.471
Time: 6	1.297	0.497	3.381
Time: 12	0.312	0.047	1.229
Site: D	1.145	0.379	3.317
Site: L	1.145	0.362	3.580
Age	1.000	0.969	1.033
Diagnosis: Lymphoma	0.686	0.175	2.357
Diagnosis: Malignant neoplasm of bronchus and lung	1.053	0.299	3.670
Diagnosis: Metastasized colorectal cancer	0.502	0.121	1.842
Diagnosis: Malignant neoplasm of pancreas	–	–	–
Diagnosis: Multiple myeloma and malignant plasma cell neoplasms	0.829	0.120	3.659
Diagnosis: Metastasized malignant neoplasm of breast	–	–	–
Diagnosis: Others	0.341	0.018	2.085

Note. Estimates shown as – indicate unstable estimates caused by complete or quasi-complete separation.

Table 9: Logistic missingness model for pattern 001010

Covariate	Odds ratio	2.5% CI	97.5% CI
Time: 3	0.339	0.093	0.993
Time: 6	0.292	0.066	0.941
Time: 12	0.219	0.034	0.821
Site: D	0.647	0.207	1.841
Site: L	0.382	0.095	1.303
Age	1.048	1.008	1.095
Diagnosis: Lymphoma	0.650	0.162	2.347
Diagnosis: Malignant neoplasm of bronchus and lung	0.217	0.011	1.417
Diagnosis: Metastasized colorectal cancer	1.068	0.305	3.740
Diagnosis: Malignant neoplasm of pancreas	–	–	–
Diagnosis: Multiple myeloma and malignant plasma cell neoplasms	0.739	0.105	3.352
Diagnosis: Metastasized malignant neoplasm of breast	1.544	0.077	10.486
Diagnosis: Others	0.450	0.023	2.873

Note. Estimates shown as – indicate unstable estimates caused by complete or quasi-complete separation.

Table 10: Logistic missingness model for pattern 000100

Covariate	Odds ratio	2.5% CI	97.5% CI
Time: 3	0.605	0.157	2.031
Time: 6	0.360	0.053	1.515
Time: 12	0.612	0.130	2.243
Site: D	1.245	0.324	4.579
Site: L	0.862	0.200	3.676
Age	0.978	0.943	1.016
Diagnosis: Lymphoma	0.270	0.014	1.751
Diagnosis: Malignant neoplasm of bronchus and lung	1.000	0.177	5.069
Diagnosis: Metastasized colorectal cancer	0.920	0.200	4.084
Diagnosis: Malignant neoplasm of pancreas	0.799	0.038	6.386
Diagnosis: Multiple myeloma and malignant plasma cell neoplasms	1.322	0.179	6.840
Diagnosis: Metastasized malignant neoplasm of breast	–	–	–
Diagnosis: Others	–	–	–

Note. Estimates shown as – indicate unstable estimates caused by complete or quasi-complete separation.

Table 11: Logistic missingness model for pattern 000101

Covariate	Odds ratio	2.5% CI	97.5% CI
Time: 3	1.478	0.323	7.562
Time: 6	1.734	0.378	8.886
Time: 12	2.440	0.590	12.030
Site: D	0.718	0.173	2.621
Site: L	0.623	0.155	2.423
Age	0.993	0.956	1.035
Diagnosis: Lymphoma	1.202	0.217	6.679
Diagnosis: Malignant neoplasm of bronchus and lung	1.184	0.140	8.308
Diagnosis: Metastasized colorectal cancer	1.201	0.201	7.247
Diagnosis: Malignant neoplasm of pancreas	1.452	0.066	13.507
Diagnosis: Multiple myeloma and malignant plasma cell neoplasms	0.895	0.043	7.328
Diagnosis: Metastasized malignant neoplasm of breast	3.355	0.156	29.717
Diagnosis: Others	2.230	0.273	14.909

Note. Estimates shown as – indicate unstable estimates caused by complete or quasi-complete separation.

Table 12: Logistic missingness model for pattern 001111

Covariate	Odds ratio	2.5% CI	97.5% CI
Time: 3	1.323	0.392	4.658
Time: 6	0.770	0.156	3.186
Time: 12	0.569	0.081	2.683
Site: D	1.125	0.337	3.866
Site: L	0.359	0.068	1.611
Age	1.014	0.972	1.064
Diagnosis: Lymphoma	4.466	0.648	88.122
Diagnosis: Malignant neoplasm of bronchus and lung	4.655	0.544	98.786
Diagnosis: Metastasized colorectal cancer	5.741	0.856	113.788
Diagnosis: Malignant neoplasm of pancreas	–	–	–
Diagnosis: Multiple myeloma and malignant plasma cell neoplasms	2.375	0.093	60.866
Diagnosis: Metastasized malignant neoplasm of breast	–	–	–
Diagnosis: Others	5.967	0.541	133.042

Note. Estimates shown as – indicate unstable estimates caused by complete or quasi-complete separation.

1.5 Main specification: tables

Table 13: Summary performance measures: main specification

Unit_Nonresponse	Missing_total	N	Method	Bias	RMSE	Coverage	Relative_Efficiency
10%	10%	50	CCA	0.086	0.322	0.976	1.000
10%	10%	50	PMM (item)	0.079	0.303	0.980	1.060
10%	10%	50	PMM (score)	0.080	0.302	0.983	1.065
10%	10%	50	RF (item)	0.078	0.301	0.981	1.069
10%	10%	50	RF (score)	0.077	0.301	0.984	1.069
10%	10%	100	CCA	0.056	0.248	0.960	1.000
10%	10%	100	PMM (item)	0.046	0.237	0.977	1.049
10%	10%	100	PMM (score)	0.046	0.235	0.977	1.056
10%	10%	100	RF (item)	0.047	0.234	0.975	1.060
10%	10%	100	RF (score)	0.047	0.234	0.977	1.060
10%	10%	200	CCA	0.017	0.167	0.971	1.000
10%	10%	200	PMM (item)	0.019	0.159	0.968	1.047
10%	10%	200	PMM (score)	0.019	0.159	0.969	1.049
10%	10%	200	RF (item)	0.021	0.159	0.969	1.049
10%	10%	200	RF (score)	0.020	0.159	0.971	1.048
10%	10%	300	CCA	0.016	0.131	0.984	1.000
10%	10%	300	PMM (item)	0.011	0.124	0.991	1.057
10%	10%	300	PMM (score)	0.011	0.124	0.989	1.056
10%	10%	300	RF (item)	0.015	0.125	0.988	1.051
10%	10%	300	RF (score)	0.014	0.124	0.988	1.052
10%	10%	400	CCA	0.032	0.095	0.991	1.000
10%	10%	400	PMM (item)	0.028	0.090	0.994	1.050
10%	10%	400	PMM (score)	0.029	0.090	0.994	1.051
10%	10%	400	RF (item)	0.033	0.092	0.991	1.030
10%	10%	400	RF (score)	0.032	0.092	0.992	1.030
10%	10%	500	CCA	0.036	0.066	1.000	1.000
10%	10%	500	PMM (item)	0.031	0.061	1.000	1.088
10%	10%	500	PMM (score)	0.031	0.060	1.000	1.098
10%	10%	500	RF (item)	0.036	0.064	1.000	1.038
10%	10%	500	RF (score)	0.036	0.064	1.000	1.042
10%	20%	50	CCA	0.088	0.351	0.964	1.000
10%	20%	50	PMM (item)	0.076	0.308	0.979	1.138
10%	20%	50	PMM (score)	0.076	0.306	0.980	1.146
10%	20%	50	RF (item)	0.072	0.305	0.979	1.151
10%	20%	50	RF (score)	0.072	0.307	0.982	1.143
10%	20%	100	CCA	0.060	0.267	0.958	1.000
10%	20%	100	PMM (item)	0.038	0.239	0.977	1.116
10%	20%	100	PMM (score)	0.039	0.238	0.976	1.121
10%	20%	100	RF (item)	0.041	0.237	0.970	1.126
10%	20%	100	RF (score)	0.038	0.236	0.975	1.129
10%	20%	200	CCA	0.019	0.183	0.967	1.000
10%	20%	200	PMM (item)	0.024	0.167	0.975	1.096
10%	20%	200	PMM (score)	0.023	0.166	0.974	1.104
10%	20%	200	RF (item)	0.026	0.167	0.967	1.100
10%	20%	200	RF (score)	0.024	0.165	0.971	1.109
10%	20%	300	CCA	0.023	0.143	0.980	1.000
10%	20%	300	PMM (item)	0.017	0.129	0.987	1.110
10%	20%	300	PMM (score)	0.017	0.128	0.984	1.117
10%	20%	300	RF (item)	0.023	0.130	0.979	1.102
10%	20%	300	RF (score)	0.022	0.129	0.984	1.107
10%	20%	400	CCA	0.032	0.105	0.987	1.000
10%	20%	400	PMM (item)	0.028	0.096	0.992	1.083
10%	20%	400	PMM (score)	0.028	0.096	0.991	1.087
10%	20%	400	RF (item)	0.035	0.098	0.987	1.061

Table 13: Summary performance measures: main specification *(continued)*

Unit_Nonresponse	Missing_total	N	Method	Bias	RMSE	Coverage	Relative_Efficiency
10%	20%	400	RF (score)	0.034	0.098	0.987	1.064
10%	20%	500	CCA	0.037	0.079	0.999	1.000
10%	20%	500	PMM (item)	0.030	0.070	1.000	1.130
10%	20%	500	PMM (score)	0.030	0.069	1.000	1.136
10%	20%	500	RF (item)	0.039	0.074	0.999	1.068
10%	20%	500	RF (score)	0.038	0.073	1.000	1.076
10%	30%	50	CCA	0.093	0.385	0.960	1.000
10%	30%	50	PMM (item)	0.078	0.315	0.978	1.220
10%	30%	50	PMM (score)	0.077	0.312	0.977	1.234
10%	30%	50	RF (item)	0.071	0.311	0.973	1.236
10%	30%	50	RF (score)	0.068	0.312	0.982	1.232
10%	30%	100	CCA	0.071	0.291	0.951	1.000
10%	30%	100	PMM (item)	0.043	0.248	0.973	1.175
10%	30%	100	PMM (score)	0.044	0.246	0.971	1.185
10%	30%	100	RF (item)	0.043	0.246	0.965	1.185
10%	30%	100	RF (score)	0.039	0.244	0.974	1.195
10%	30%	200	CCA	0.022	0.199	0.962	1.000
10%	30%	200	PMM (item)	0.028	0.172	0.977	1.160
10%	30%	200	PMM (score)	0.029	0.172	0.975	1.163
10%	30%	200	RF (item)	0.030	0.172	0.968	1.160
10%	30%	200	RF (score)	0.028	0.170	0.973	1.174
10%	30%	300	CCA	0.028	0.158	0.979	1.000
10%	30%	300	PMM (item)	0.024	0.136	0.987	1.158
10%	30%	300	PMM (score)	0.023	0.135	0.986	1.171
10%	30%	300	RF (item)	0.030	0.137	0.977	1.155
10%	30%	300	RF (score)	0.028	0.136	0.980	1.163
10%	30%	400	CCA	0.035	0.119	0.985	1.000
10%	30%	400	PMM (item)	0.030	0.103	0.989	1.161
10%	30%	400	PMM (score)	0.029	0.102	0.993	1.170
10%	30%	400	RF (item)	0.040	0.107	0.983	1.117
10%	30%	400	RF (score)	0.038	0.105	0.986	1.133
10%	30%	500	CCA	0.042	0.095	0.995	1.000
10%	30%	500	PMM (item)	0.031	0.076	0.999	1.235
10%	30%	500	PMM (score)	0.031	0.076	0.999	1.247
10%	30%	500	RF (item)	0.045	0.083	0.996	1.136
10%	30%	500	RF (score)	0.042	0.082	0.997	1.152
10%	50%	50	CCA	0.115	0.494	0.922	1.000
10%	50%	50	PMM (item)	0.080	0.334	0.968	1.477
10%	50%	50	PMM (score)	0.078	0.329	0.969	1.503
10%	50%	50	RF (item)	0.067	0.332	0.957	1.487
10%	50%	50	RF (score)	0.062	0.337	0.973	1.468
10%	50%	100	CCA	0.091	0.357	0.928	1.000
10%	50%	100	PMM (item)	0.059	0.263	0.962	1.356
10%	50%	100	PMM (score)	0.062	0.261	0.960	1.365
10%	50%	100	RF (item)	0.042	0.252	0.950	1.413
10%	50%	100	RF (score)	0.041	0.254	0.967	1.406
10%	50%	200	CCA	0.046	0.246	0.949	1.000
10%	50%	200	PMM (item)	0.055	0.192	0.969	1.279
10%	50%	200	PMM (score)	0.056	0.190	0.970	1.294
10%	50%	200	RF (item)	0.052	0.189	0.954	1.304
10%	50%	200	RF (score)	0.050	0.185	0.968	1.328
10%	50%	300	CCA	0.052	0.197	0.962	1.000
10%	50%	300	PMM (item)	0.044	0.150	0.979	1.319
10%	50%	300	PMM (score)	0.045	0.148	0.978	1.333
10%	50%	300	RF (item)	0.050	0.150	0.968	1.319
10%	50%	300	RF (score)	0.047	0.147	0.971	1.345
10%	50%	400	CCA	0.054	0.156	0.974	1.000

Table 13: Summary performance measures: main specification *(continued)*

Unit_Nonresponse	Missing_total	N	Method	Bias	RMSE	Coverage	Relative_Efficiency
10%	50%	400	PMM (item)	0.044	0.122	0.984	1.280
10%	50%	400	PMM (score)	0.044	0.122	0.984	1.284
10%	50%	400	RF (item)	0.056	0.126	0.968	1.236
10%	50%	400	RF (score)	0.053	0.124	0.974	1.260
10%	50%	500	CCA	0.060	0.131	0.981	1.000
10%	50%	500	PMM (item)	0.041	0.097	0.993	1.352
10%	50%	500	PMM (score)	0.042	0.096	0.995	1.369
10%	50%	500	RF (item)	0.057	0.103	0.978	1.278
10%	50%	500	RF (score)	0.055	0.101	0.984	1.296
20%	10%	50	CCA	0.086	0.325	0.974	1.000
20%	10%	50	PMM (item)	0.078	0.303	0.981	1.070
20%	10%	50	PMM (score)	0.079	0.303	0.982	1.072
20%	10%	50	RF (item)	0.076	0.302	0.981	1.076
20%	10%	50	RF (score)	0.076	0.302	0.981	1.074
20%	10%	100	CCA	0.055	0.245	0.962	1.000
20%	10%	100	PMM (item)	0.045	0.235	0.977	1.044
20%	10%	100	PMM (score)	0.045	0.234	0.977	1.050
20%	10%	100	RF (item)	0.046	0.234	0.976	1.048
20%	10%	100	RF (score)	0.045	0.233	0.977	1.052
20%	10%	200	CCA	0.018	0.166	0.972	1.000
20%	10%	200	PMM (item)	0.019	0.158	0.976	1.045
20%	10%	200	PMM (score)	0.019	0.158	0.975	1.048
20%	10%	200	RF (item)	0.019	0.158	0.973	1.050
20%	10%	200	RF (score)	0.019	0.158	0.976	1.051
20%	10%	300	CCA	0.017	0.132	0.983	1.000
20%	10%	300	PMM (item)	0.012	0.125	0.987	1.052
20%	10%	300	PMM (score)	0.012	0.125	0.987	1.056
20%	10%	300	RF (item)	0.016	0.126	0.984	1.047
20%	10%	300	RF (score)	0.016	0.125	0.986	1.054
20%	10%	400	CCA	0.031	0.095	0.990	1.000
20%	10%	400	PMM (item)	0.029	0.091	0.994	1.049
20%	10%	400	PMM (score)	0.029	0.091	0.994	1.048
20%	10%	400	RF (item)	0.032	0.092	0.991	1.037
20%	10%	400	RF (score)	0.032	0.092	0.993	1.037
20%	10%	500	CCA	0.036	0.067	1.000	1.000
20%	10%	500	PMM (item)	0.031	0.062	1.000	1.083
20%	10%	500	PMM (score)	0.031	0.061	1.000	1.088
20%	10%	500	RF (item)	0.036	0.064	1.000	1.037
20%	10%	500	RF (score)	0.036	0.064	1.000	1.040
20%	20%	50	CCA	0.088	0.354	0.971	1.000
20%	20%	50	PMM (item)	0.071	0.307	0.982	1.152
20%	20%	50	PMM (score)	0.072	0.307	0.980	1.153
20%	20%	50	RF (item)	0.070	0.304	0.980	1.164
20%	20%	50	RF (score)	0.070	0.306	0.983	1.158
20%	20%	100	CCA	0.060	0.266	0.956	1.000
20%	20%	100	PMM (item)	0.042	0.239	0.978	1.116
20%	20%	100	PMM (score)	0.043	0.238	0.978	1.118
20%	20%	100	RF (item)	0.044	0.238	0.970	1.122
20%	20%	100	RF (score)	0.042	0.237	0.977	1.123
20%	20%	200	CCA	0.021	0.180	0.966	1.000
20%	20%	200	PMM (item)	0.026	0.167	0.976	1.083
20%	20%	200	PMM (score)	0.026	0.166	0.973	1.086
20%	20%	200	RF (item)	0.028	0.166	0.969	1.086
20%	20%	200	RF (score)	0.026	0.165	0.971	1.093
20%	20%	300	CCA	0.021	0.143	0.981	1.000
20%	20%	300	PMM (item)	0.016	0.130	0.989	1.096
20%	20%	300	PMM (score)	0.016	0.129	0.989	1.107

Table 13: Summary performance measures: main specification *(continued)*

Unit_Nonresponse	Missing_total	N	Method	Bias	RMSE	Coverage	Relative_Efficiency
20%	20%	300	RF (item)	0.022	0.131	0.985	1.093
20%	20%	300	RF (score)	0.020	0.129	0.987	1.102
20%	20%	400	CCA	0.034	0.107	0.986	1.000
20%	20%	400	PMM (item)	0.030	0.097	0.991	1.095
20%	20%	400	PMM (score)	0.029	0.097	0.991	1.103
20%	20%	400	RF (item)	0.037	0.099	0.987	1.073
20%	20%	400	RF (score)	0.035	0.099	0.990	1.082
20%	20%	500	CCA	0.038	0.077	0.999	1.000
20%	20%	500	PMM (item)	0.030	0.068	1.000	1.135
20%	20%	500	PMM (score)	0.030	0.069	1.000	1.127
20%	20%	500	RF (item)	0.040	0.073	0.999	1.061
20%	20%	500	RF (score)	0.038	0.072	0.999	1.075
20%	30%	50	CCA	0.095	0.393	0.953	1.000
20%	30%	50	PMM (item)	0.071	0.317	0.978	1.239
20%	30%	50	PMM (score)	0.073	0.315	0.976	1.247
20%	30%	50	RF (item)	0.066	0.312	0.976	1.260
20%	30%	50	RF (score)	0.065	0.313	0.984	1.253
20%	30%	100	CCA	0.070	0.288	0.950	1.000
20%	30%	100	PMM (item)	0.042	0.246	0.971	1.170
20%	30%	100	PMM (score)	0.044	0.245	0.969	1.175
20%	30%	100	RF (item)	0.041	0.244	0.959	1.181
20%	30%	100	RF (score)	0.041	0.242	0.969	1.192
20%	30%	200	CCA	0.021	0.197	0.960	1.000
20%	30%	200	PMM (item)	0.030	0.172	0.974	1.146
20%	30%	200	PMM (score)	0.031	0.170	0.977	1.156
20%	30%	200	RF (item)	0.033	0.173	0.963	1.135
20%	30%	200	RF (score)	0.032	0.171	0.970	1.152
20%	30%	300	CCA	0.027	0.156	0.975	1.000
20%	30%	300	PMM (item)	0.024	0.136	0.985	1.143
20%	30%	300	PMM (score)	0.024	0.135	0.987	1.156
20%	30%	300	RF (item)	0.030	0.135	0.978	1.149
20%	30%	300	RF (score)	0.028	0.134	0.982	1.165
20%	30%	400	CCA	0.035	0.119	0.983	1.000
20%	30%	400	PMM (item)	0.033	0.106	0.989	1.124
20%	30%	400	PMM (score)	0.033	0.105	0.988	1.134
20%	30%	400	RF (item)	0.041	0.108	0.984	1.099
20%	30%	400	RF (score)	0.040	0.107	0.988	1.110
20%	30%	500	CCA	0.042	0.094	0.995	1.000
20%	30%	500	PMM (item)	0.030	0.078	0.999	1.213
20%	30%	500	PMM (score)	0.030	0.077	0.999	1.218
20%	30%	500	RF (item)	0.044	0.083	0.996	1.133
20%	30%	500	RF (score)	0.042	0.082	0.998	1.140
20%	50%	50	CCA	0.117	0.499	0.921	1.000
20%	50%	50	PMM (item)	0.077	0.336	0.968	1.486
20%	50%	50	PMM (score)	0.074	0.327	0.967	1.527
20%	50%	50	RF (item)	0.063	0.329	0.959	1.516
20%	50%	50	RF (score)	0.061	0.331	0.977	1.506
20%	50%	100	CCA	0.090	0.359	0.925	1.000
20%	50%	100	PMM (item)	0.061	0.268	0.964	1.339
20%	50%	100	PMM (score)	0.063	0.265	0.963	1.354
20%	50%	100	RF (item)	0.045	0.257	0.946	1.393
20%	50%	100	RF (score)	0.045	0.257	0.968	1.396
20%	50%	200	CCA	0.047	0.249	0.947	1.000
20%	50%	200	PMM (item)	0.053	0.192	0.969	1.294
20%	50%	200	PMM (score)	0.054	0.189	0.972	1.315
20%	50%	200	RF (item)	0.053	0.190	0.952	1.308
20%	50%	200	RF (score)	0.050	0.187	0.968	1.330

Table 13: Summary performance measures: main specification *(continued)*

Unit_Nonresponse	Missing_total	N	Method	Bias	RMSE	Coverage	Relative_Efficiency
20%	50%	300	CCA	0.053	0.199	0.959	1.000
20%	50%	300	PMM (item)	0.043	0.150	0.979	1.328
20%	50%	300	PMM (score)	0.044	0.148	0.980	1.340
20%	50%	300	RF (item)	0.048	0.150	0.965	1.322
20%	50%	300	RF (score)	0.046	0.148	0.973	1.346
20%	50%	400	CCA	0.055	0.158	0.967	1.000
20%	50%	400	PMM (item)	0.044	0.125	0.983	1.271
20%	50%	400	PMM (score)	0.043	0.123	0.984	1.282
20%	50%	400	RF (item)	0.054	0.127	0.964	1.243
20%	50%	400	RF (score)	0.051	0.125	0.976	1.266
20%	50%	500	CCA	0.060	0.129	0.985	1.000
20%	50%	500	PMM (item)	0.040	0.096	0.994	1.349
20%	50%	500	PMM (score)	0.041	0.095	0.996	1.365
20%	50%	500	RF (item)	0.058	0.102	0.984	1.266
20%	50%	500	RF (score)	0.055	0.101	0.990	1.288
30%	10%	50	CCA	0.081	0.319	0.981	1.000
30%	10%	50	PMM (item)	0.075	0.300	0.984	1.062
30%	10%	50	PMM (score)	0.075	0.299	0.985	1.065
30%	10%	50	RF (item)	0.074	0.301	0.983	1.061
30%	10%	50	RF (score)	0.073	0.299	0.988	1.066
30%	10%	100	CCA	0.057	0.248	0.963	1.000
30%	10%	100	PMM (item)	0.046	0.235	0.978	1.053
30%	10%	100	PMM (score)	0.046	0.235	0.979	1.055
30%	10%	100	RF (item)	0.048	0.235	0.976	1.056
30%	10%	100	RF (score)	0.048	0.234	0.977	1.059
30%	10%	200	CCA	0.017	0.166	0.970	1.000
30%	10%	200	PMM (item)	0.019	0.159	0.973	1.042
30%	10%	200	PMM (score)	0.019	0.159	0.972	1.045
30%	10%	200	RF (item)	0.020	0.160	0.970	1.040
30%	10%	200	RF (score)	0.020	0.160	0.971	1.039
30%	10%	300	CCA	0.015	0.130	0.986	1.000
30%	10%	300	PMM (item)	0.011	0.124	0.991	1.052
30%	10%	300	PMM (score)	0.011	0.123	0.993	1.058
30%	10%	300	RF (item)	0.015	0.124	0.988	1.045
30%	10%	300	RF (score)	0.014	0.124	0.989	1.046
30%	10%	400	CCA	0.030	0.094	0.990	1.000
30%	10%	400	PMM (item)	0.027	0.090	0.994	1.044
30%	10%	400	PMM (score)	0.027	0.090	0.993	1.045
30%	10%	400	RF (item)	0.030	0.091	0.992	1.034
30%	10%	400	RF (score)	0.030	0.091	0.993	1.037
30%	10%	500	CCA	0.035	0.066	1.000	1.000
30%	10%	500	PMM (item)	0.030	0.061	1.000	1.086
30%	10%	500	PMM (score)	0.030	0.061	1.000	1.088
30%	10%	500	RF (item)	0.036	0.064	1.000	1.034
30%	10%	500	RF (score)	0.035	0.063	1.000	1.044
30%	20%	50	CCA	0.088	0.347	0.970	1.000
30%	20%	50	PMM (item)	0.074	0.307	0.980	1.130
30%	20%	50	PMM (score)	0.075	0.305	0.982	1.136
30%	20%	50	RF (item)	0.073	0.302	0.980	1.147
30%	20%	50	RF (score)	0.069	0.301	0.986	1.150
30%	20%	100	CCA	0.060	0.265	0.957	1.000
30%	20%	100	PMM (item)	0.043	0.239	0.975	1.110
30%	20%	100	PMM (score)	0.043	0.237	0.976	1.117
30%	20%	100	RF (item)	0.044	0.237	0.970	1.117
30%	20%	100	RF (score)	0.042	0.237	0.973	1.118
30%	20%	200	CCA	0.021	0.180	0.967	1.000
30%	20%	200	PMM (item)	0.025	0.166	0.976	1.088

Table 13: Summary performance measures: main specification *(continued)*

Unit_Nonresponse	Missing_total	N	Method	Bias	RMSE	Coverage	Relative_Efficiency
30%	20%	200	PMM (score)	0.024	0.165	0.975	1.093
30%	20%	200	RF (item)	0.027	0.165	0.972	1.095
30%	20%	200	RF (score)	0.025	0.164	0.974	1.098
30%	20%	300	CCA	0.021	0.142	0.979	1.000
30%	20%	300	PMM (item)	0.015	0.129	0.986	1.094
30%	20%	300	PMM (score)	0.015	0.129	0.985	1.099
30%	20%	300	RF (item)	0.022	0.130	0.982	1.091
30%	20%	300	RF (score)	0.020	0.129	0.986	1.098
30%	20%	400	CCA	0.030	0.106	0.987	1.000
30%	20%	400	PMM (item)	0.028	0.098	0.995	1.089
30%	20%	400	PMM (score)	0.027	0.097	0.994	1.096
30%	20%	400	RF (item)	0.033	0.100	0.988	1.064
30%	20%	400	RF (score)	0.032	0.099	0.991	1.075
30%	20%	500	CCA	0.039	0.079	0.998	1.000
30%	20%	500	PMM (item)	0.030	0.069	1.000	1.142
30%	20%	500	PMM (score)	0.031	0.069	1.000	1.140
30%	20%	500	RF (item)	0.041	0.074	1.000	1.067
30%	20%	500	RF (score)	0.039	0.073	1.000	1.077
30%	30%	50	CCA	0.097	0.386	0.954	1.000
30%	30%	50	PMM (item)	0.077	0.316	0.979	1.222
30%	30%	50	PMM (score)	0.077	0.313	0.976	1.230
30%	30%	50	RF (item)	0.069	0.312	0.976	1.238
30%	30%	50	RF (score)	0.069	0.314	0.981	1.226
30%	30%	100	CCA	0.065	0.291	0.948	1.000
30%	30%	100	PMM (item)	0.042	0.246	0.973	1.183
30%	30%	100	PMM (score)	0.043	0.245	0.973	1.189
30%	30%	100	RF (item)	0.039	0.241	0.966	1.207
30%	30%	100	RF (score)	0.037	0.241	0.974	1.208
30%	30%	200	CCA	0.023	0.199	0.956	1.000
30%	30%	200	PMM (item)	0.032	0.175	0.972	1.138
30%	30%	200	PMM (score)	0.033	0.173	0.974	1.150
30%	30%	200	RF (item)	0.032	0.174	0.964	1.141
30%	30%	200	RF (score)	0.031	0.171	0.973	1.160
30%	30%	300	CCA	0.030	0.157	0.973	1.000
30%	30%	300	PMM (item)	0.026	0.137	0.983	1.141
30%	30%	300	PMM (score)	0.025	0.135	0.985	1.158
30%	30%	300	RF (item)	0.033	0.135	0.974	1.157
30%	30%	300	RF (score)	0.031	0.135	0.978	1.165
30%	30%	400	CCA	0.035	0.120	0.980	1.000
30%	30%	400	PMM (item)	0.031	0.105	0.989	1.145
30%	30%	400	PMM (score)	0.030	0.104	0.989	1.157
30%	30%	400	RF (item)	0.040	0.107	0.980	1.119
30%	30%	400	RF (score)	0.038	0.106	0.983	1.132
30%	30%	500	CCA	0.041	0.092	0.993	1.000
30%	30%	500	PMM (item)	0.030	0.077	0.999	1.203
30%	30%	500	PMM (score)	0.030	0.075	0.999	1.222
30%	30%	500	RF (item)	0.043	0.081	0.997	1.140
30%	30%	500	RF (score)	0.041	0.080	0.998	1.148
30%	50%	50	CCA	0.117	0.494	0.918	1.000
30%	50%	50	PMM (item)	0.077	0.341	0.968	1.449
30%	50%	50	PMM (score)	0.075	0.335	0.965	1.475
30%	50%	50	RF (item)	0.062	0.331	0.958	1.491
30%	50%	50	RF (score)	0.063	0.335	0.976	1.475
30%	50%	100	CCA	0.092	0.356	0.938	1.000
30%	50%	100	PMM (item)	0.061	0.261	0.966	1.364
30%	50%	100	PMM (score)	0.062	0.259	0.966	1.372
30%	50%	100	RF (item)	0.045	0.250	0.955	1.423

Table 13: Summary performance measures: main specification *(continued)*

Unit_Nonresponse	Missing_total	N	Method	Bias	RMSE	Coverage	Relative_Efficiency
30%	50%	100	RF (score)	0.045	0.249	0.967	1.426
30%	50%	200	CCA	0.043	0.250	0.946	1.000
30%	50%	200	PMM (item)	0.046	0.194	0.970	1.290
30%	50%	200	PMM (score)	0.048	0.191	0.970	1.307
30%	50%	200	RF (item)	0.047	0.190	0.950	1.317
30%	50%	200	RF (score)	0.044	0.187	0.967	1.337
30%	50%	300	CCA	0.050	0.198	0.961	1.000
30%	50%	300	PMM (item)	0.042	0.151	0.979	1.311
30%	50%	300	PMM (score)	0.043	0.149	0.982	1.325
30%	50%	300	RF (item)	0.049	0.150	0.959	1.317
30%	50%	300	RF (score)	0.045	0.148	0.972	1.339
30%	50%	400	CCA	0.053	0.156	0.971	1.000
30%	50%	400	PMM (item)	0.044	0.124	0.984	1.257
30%	50%	400	PMM (score)	0.044	0.122	0.985	1.271
30%	50%	400	RF (item)	0.055	0.126	0.965	1.240
30%	50%	400	RF (score)	0.051	0.123	0.973	1.269
30%	50%	500	CCA	0.055	0.128	0.982	1.000
30%	50%	500	PMM (item)	0.039	0.098	0.993	1.309
30%	50%	500	PMM (score)	0.039	0.095	0.993	1.340
30%	50%	500	RF (item)	0.056	0.104	0.981	1.231
30%	50%	500	RF (score)	0.053	0.102	0.985	1.253
50%	10%	50	CCA	0.088	0.325	0.977	1.000
50%	10%	50	PMM (item)	0.081	0.303	0.984	1.071
50%	10%	50	PMM (score)	0.081	0.302	0.983	1.074
50%	10%	50	RF (item)	0.080	0.302	0.981	1.074
50%	10%	50	RF (score)	0.079	0.303	0.984	1.072
50%	10%	100	CCA	0.055	0.248	0.962	1.000
50%	10%	100	PMM (item)	0.044	0.236	0.977	1.050
50%	10%	100	PMM (score)	0.044	0.235	0.977	1.053
50%	10%	100	RF (item)	0.046	0.234	0.974	1.059
50%	10%	100	RF (score)	0.045	0.234	0.977	1.057
50%	10%	200	CCA	0.015	0.167	0.967	1.000
50%	10%	200	PMM (item)	0.017	0.160	0.972	1.043
50%	10%	200	PMM (score)	0.018	0.160	0.971	1.046
50%	10%	200	RF (item)	0.018	0.160	0.971	1.046
50%	10%	200	RF (score)	0.018	0.159	0.972	1.047
50%	10%	300	CCA	0.017	0.132	0.982	1.000
50%	10%	300	PMM (item)	0.012	0.125	0.987	1.056
50%	10%	300	PMM (score)	0.013	0.125	0.987	1.055
50%	10%	300	RF (item)	0.016	0.126	0.986	1.052
50%	10%	300	RF (score)	0.015	0.126	0.985	1.051
50%	10%	400	CCA	0.033	0.095	0.989	1.000
50%	10%	400	PMM (item)	0.030	0.091	0.994	1.040
50%	10%	400	PMM (score)	0.030	0.091	0.992	1.041
50%	10%	400	RF (item)	0.033	0.092	0.991	1.036
50%	10%	400	RF (score)	0.032	0.092	0.993	1.038
50%	10%	500	CCA	0.036	0.067	1.000	1.000
50%	10%	500	PMM (item)	0.031	0.061	1.000	1.091
50%	10%	500	PMM (score)	0.031	0.061	1.000	1.094
50%	10%	500	RF (item)	0.036	0.064	1.000	1.042
50%	10%	500	RF (score)	0.035	0.063	1.000	1.048
50%	20%	50	CCA	0.089	0.354	0.969	1.000
50%	20%	50	PMM (item)	0.074	0.307	0.984	1.153
50%	20%	50	PMM (score)	0.074	0.305	0.983	1.158
50%	20%	50	RF (item)	0.072	0.304	0.980	1.162
50%	20%	50	RF (score)	0.073	0.304	0.988	1.163
50%	20%	100	CCA	0.060	0.266	0.956	1.000

Table 13: Summary performance measures: main specification *(continued)*

Unit_Nonresponse	Missing_total	N	Method	Bias	RMSE	Coverage	Relative_Efficiency
50%	20%	100	PMM (item)	0.042	0.242	0.976	1.098
50%	20%	100	PMM (score)	0.042	0.241	0.976	1.102
50%	20%	100	RF (item)	0.043	0.237	0.970	1.119
50%	20%	100	RF (score)	0.041	0.237	0.976	1.119
50%	20%	200	CCA	0.021	0.179	0.970	1.000
50%	20%	200	PMM (item)	0.025	0.165	0.975	1.085
50%	20%	200	PMM (score)	0.025	0.165	0.976	1.086
50%	20%	200	RF (item)	0.027	0.165	0.970	1.081
50%	20%	200	RF (score)	0.026	0.164	0.976	1.089
50%	20%	300	CCA	0.020	0.143	0.977	1.000
50%	20%	300	PMM (item)	0.016	0.130	0.991	1.104
50%	20%	300	PMM (score)	0.016	0.129	0.990	1.113
50%	20%	300	RF (item)	0.021	0.130	0.983	1.102
50%	20%	300	RF (score)	0.019	0.129	0.985	1.110
50%	20%	400	CCA	0.034	0.105	0.989	1.000
50%	20%	400	PMM (item)	0.029	0.098	0.991	1.078
50%	20%	400	PMM (score)	0.029	0.097	0.994	1.085
50%	20%	400	RF (item)	0.036	0.100	0.988	1.058
50%	20%	400	RF (score)	0.035	0.099	0.991	1.067
50%	20%	500	CCA	0.038	0.079	0.998	1.000
50%	20%	500	PMM (item)	0.030	0.069	1.000	1.149
50%	20%	500	PMM (score)	0.030	0.068	1.000	1.160
50%	20%	500	RF (item)	0.040	0.073	0.999	1.079
50%	20%	500	RF (score)	0.038	0.073	0.999	1.088
50%	30%	50	CCA	0.094	0.382	0.960	1.000
50%	30%	50	PMM (item)	0.076	0.316	0.978	1.211
50%	30%	50	PMM (score)	0.077	0.313	0.978	1.222
50%	30%	50	RF (item)	0.070	0.308	0.972	1.240
50%	30%	50	RF (score)	0.068	0.310	0.982	1.234
50%	30%	100	CCA	0.071	0.288	0.953	1.000
50%	30%	100	PMM (item)	0.042	0.248	0.975	1.162
50%	30%	100	PMM (score)	0.042	0.246	0.974	1.170
50%	30%	100	RF (item)	0.041	0.242	0.966	1.189
50%	30%	100	RF (score)	0.038	0.241	0.977	1.195
50%	30%	200	CCA	0.024	0.199	0.956	1.000
50%	30%	200	PMM (item)	0.030	0.174	0.976	1.145
50%	30%	200	PMM (score)	0.031	0.172	0.974	1.155
50%	30%	200	RF (item)	0.031	0.173	0.963	1.150
50%	30%	200	RF (score)	0.030	0.171	0.972	1.162
50%	30%	300	CCA	0.029	0.157	0.974	1.000
50%	30%	300	PMM (item)	0.024	0.137	0.985	1.153
50%	30%	300	PMM (score)	0.024	0.135	0.984	1.164
50%	30%	300	RF (item)	0.031	0.137	0.974	1.147
50%	30%	300	RF (score)	0.030	0.136	0.978	1.154
50%	30%	400	CCA	0.032	0.120	0.981	1.000
50%	30%	400	PMM (item)	0.031	0.105	0.989	1.138
50%	30%	400	PMM (score)	0.031	0.104	0.988	1.151
50%	30%	400	RF (item)	0.040	0.107	0.982	1.121
50%	30%	400	RF (score)	0.038	0.106	0.984	1.132
50%	30%	500	CCA	0.041	0.093	0.993	1.000
50%	30%	500	PMM (item)	0.032	0.077	0.998	1.206
50%	30%	500	PMM (score)	0.031	0.076	0.999	1.217
50%	30%	500	RF (item)	0.044	0.083	0.995	1.121
50%	30%	500	RF (score)	0.042	0.081	0.996	1.144
50%	50%	50	CCA	0.117	0.495	0.918	1.000
50%	50%	50	PMM (item)	0.078	0.332	0.967	1.490
50%	50%	50	PMM (score)	0.078	0.327	0.965	1.514

Table 13: Summary performance measures: main specification *(continued)*

Unit_Nonresponse	Missing_total	N	Method	Bias	RMSE	Coverage	Relative_Efficiency
50%	50%	50	RF (item)	0.067	0.327	0.958	1.512
50%	50%	50	RF (score)	0.064	0.331	0.977	1.496
50%	50%	100	CCA	0.083	0.355	0.933	1.000
50%	50%	100	PMM (item)	0.057	0.269	0.963	1.322
50%	50%	100	PMM (score)	0.058	0.263	0.964	1.349
50%	50%	100	RF (item)	0.044	0.258	0.948	1.376
50%	50%	100	RF (score)	0.041	0.254	0.966	1.396
50%	50%	200	CCA	0.043	0.245	0.951	1.000
50%	50%	200	PMM (item)	0.048	0.191	0.972	1.281
50%	50%	200	PMM (score)	0.050	0.189	0.973	1.300
50%	50%	200	RF (item)	0.047	0.186	0.958	1.319
50%	50%	200	RF (score)	0.045	0.183	0.970	1.340
50%	50%	300	CCA	0.049	0.197	0.959	1.000
50%	50%	300	PMM (item)	0.046	0.153	0.978	1.284
50%	50%	300	PMM (score)	0.046	0.151	0.978	1.302
50%	50%	300	RF (item)	0.052	0.153	0.959	1.286
50%	50%	300	RF (score)	0.049	0.150	0.971	1.311
50%	50%	400	CCA	0.051	0.160	0.969	1.000
50%	50%	400	PMM (item)	0.044	0.124	0.984	1.290
50%	50%	400	PMM (score)	0.044	0.122	0.984	1.308
50%	50%	400	RF (item)	0.056	0.127	0.967	1.256
50%	50%	400	RF (score)	0.052	0.126	0.971	1.272
50%	50%	500	CCA	0.059	0.129	0.984	1.000
50%	50%	500	PMM (item)	0.043	0.098	0.995	1.324
50%	50%	500	PMM (score)	0.044	0.097	0.993	1.333
50%	50%	500	RF (item)	0.060	0.105	0.981	1.233
50%	50%	500	RF (score)	0.057	0.102	0.988	1.261

Table 14: Variance-related measures: main specification

Unit_Nonresponse	Missing_total	N	Method	Empirical_Var	Estimated_Var	True_Var	Var_Ratio
10%	10%	50	CCA	0.0251	0.0360	0.0323	1.437
10%	10%	50	PMM (item)	0.0221	0.0341	0.0323	1.544
10%	10%	50	PMM (score)	0.0219	0.0338	0.0323	1.546
10%	10%	50	RF (item)	0.0219	0.0328	0.0323	1.502
10%	10%	50	RF (score)	0.0218	0.0344	0.0323	1.576
10%	10%	100	CCA	0.0154	0.0166	0.0149	1.082
10%	10%	100	PMM (item)	0.0140	0.0166	0.0149	1.187
10%	10%	100	PMM (score)	0.0138	0.0165	0.0149	1.195
10%	10%	100	RF (item)	0.0137	0.0158	0.0149	1.158
10%	10%	100	RF (score)	0.0137	0.0163	0.0149	1.195
10%	10%	200	CCA	0.0070	0.0088	0.0079	1.268
10%	10%	200	PMM (item)	0.0063	0.0088	0.0079	1.394
10%	10%	200	PMM (score)	0.0063	0.0088	0.0079	1.391
10%	10%	200	RF (item)	0.0063	0.0084	0.0079	1.340
10%	10%	200	RF (score)	0.0063	0.0087	0.0079	1.369
10%	10%	300	CCA	0.0043	0.0058	0.0052	1.363
10%	10%	300	PMM (item)	0.0038	0.0059	0.0052	1.533
10%	10%	300	PMM (score)	0.0038	0.0059	0.0052	1.527
10%	10%	300	RF (item)	0.0039	0.0056	0.0052	1.461
10%	10%	300	RF (score)	0.0039	0.0057	0.0052	1.488
10%	10%	400	CCA	0.0022	0.0043	0.0038	1.922
10%	10%	400	PMM (item)	0.0020	0.0043	0.0038	2.130
10%	10%	400	PMM (score)	0.0020	0.0043	0.0038	2.127
10%	10%	400	RF (item)	0.0021	0.0042	0.0038	2.010
10%	10%	400	RF (score)	0.0021	0.0042	0.0038	2.037
10%	10%	500	CCA	0.0010	0.0034	0.0031	3.293
10%	10%	500	PMM (item)	0.0009	0.0034	0.0031	3.830
10%	10%	500	PMM (score)	0.0009	0.0034	0.0031	3.883
10%	10%	500	RF (item)	0.0009	0.0033	0.0031	3.591
10%	10%	500	RF (score)	0.0009	0.0034	0.0031	3.633
10%	20%	50	CCA	0.0298	0.0409	0.0323	1.369
10%	20%	50	PMM (item)	0.0227	0.0355	0.0323	1.564
10%	20%	50	PMM (score)	0.0223	0.0348	0.0323	1.559
10%	20%	50	RF (item)	0.0224	0.0332	0.0323	1.484
10%	20%	50	RF (score)	0.0227	0.0365	0.0323	1.608
10%	20%	100	CCA	0.0178	0.0189	0.0149	1.064
10%	20%	100	PMM (item)	0.0142	0.0179	0.0149	1.258
10%	20%	100	PMM (score)	0.0141	0.0177	0.0149	1.254
10%	20%	100	RF (item)	0.0140	0.0163	0.0149	1.162
10%	20%	100	RF (score)	0.0139	0.0174	0.0149	1.248
10%	20%	200	CCA	0.0084	0.0100	0.0079	1.191
10%	20%	200	PMM (item)	0.0070	0.0096	0.0079	1.382
10%	20%	200	PMM (score)	0.0069	0.0095	0.0079	1.389
10%	20%	200	RF (item)	0.0069	0.0087	0.0079	1.272
10%	20%	200	RF (score)	0.0068	0.0092	0.0079	1.358
10%	20%	300	CCA	0.0051	0.0066	0.0052	1.297
10%	20%	300	PMM (item)	0.0042	0.0064	0.0052	1.553
10%	20%	300	PMM (score)	0.0041	0.0064	0.0052	1.562
10%	20%	300	RF (item)	0.0041	0.0059	0.0052	1.424
10%	20%	300	RF (score)	0.0041	0.0061	0.0052	1.486
10%	20%	400	CCA	0.0027	0.0049	0.0038	1.802
10%	20%	400	PMM (item)	0.0023	0.0047	0.0038	2.045
10%	20%	400	PMM (score)	0.0023	0.0047	0.0038	2.045
10%	20%	400	RF (item)	0.0023	0.0044	0.0038	1.886
10%	20%	400	RF (score)	0.0023	0.0045	0.0038	1.938
10%	20%	500	CCA	0.0015	0.0039	0.0031	2.625
10%	20%	500	PMM (item)	0.0012	0.0038	0.0031	3.170

Table 14: Variance-related measures: main specification *(continued)*

Unit_Nonresponse	Missing_total	N	Method	Empirical_Var	Estimated_Var	True_Var	Var_Ratio
10%	20%	500	PMM (score)	0.0012	0.0037	0.0031	3.184
10%	20%	500	RF (item)	0.0012	0.0035	0.0031	2.911
10%	20%	500	RF (score)	0.0012	0.0036	0.0031	2.993
10%	30%	50	CCA	0.0361	0.0466	0.0323	1.288
10%	30%	50	PMM (item)	0.0236	0.0365	0.0323	1.547
10%	30%	50	PMM (score)	0.0230	0.0355	0.0323	1.538
10%	30%	50	RF (item)	0.0233	0.0328	0.0323	1.409
10%	30%	50	RF (score)	0.0235	0.0382	0.0323	1.623
10%	30%	100	CCA	0.0212	0.0219	0.0149	1.033
10%	30%	100	PMM (item)	0.0152	0.0194	0.0149	1.279
10%	30%	100	PMM (score)	0.0149	0.0191	0.0149	1.281
10%	30%	100	RF (item)	0.0150	0.0165	0.0149	1.099
10%	30%	100	RF (score)	0.0148	0.0186	0.0149	1.256
10%	30%	200	CCA	0.0099	0.0115	0.0079	1.157
10%	30%	200	PMM (item)	0.0074	0.0105	0.0079	1.427
10%	30%	200	PMM (score)	0.0073	0.0104	0.0079	1.416
10%	30%	200	RF (item)	0.0073	0.0091	0.0079	1.242
10%	30%	200	RF (score)	0.0071	0.0098	0.0079	1.372
10%	30%	300	CCA	0.0062	0.0076	0.0052	1.230
10%	30%	300	PMM (item)	0.0046	0.0071	0.0052	1.534
10%	30%	300	PMM (score)	0.0045	0.0070	0.0052	1.542
10%	30%	300	RF (item)	0.0045	0.0061	0.0052	1.356
10%	30%	300	RF (score)	0.0045	0.0065	0.0052	1.449
10%	30%	400	CCA	0.0035	0.0056	0.0038	1.595
10%	30%	400	PMM (item)	0.0026	0.0052	0.0038	1.988
10%	30%	400	PMM (score)	0.0026	0.0052	0.0038	1.998
10%	30%	400	RF (item)	0.0027	0.0046	0.0038	1.723
10%	30%	400	RF (score)	0.0026	0.0048	0.0038	1.842
10%	30%	500	CCA	0.0021	0.0045	0.0031	2.095
10%	30%	500	PMM (item)	0.0014	0.0042	0.0031	2.877
10%	30%	500	PMM (score)	0.0014	0.0041	0.0031	2.896
10%	30%	500	RF (item)	0.0015	0.0037	0.0031	2.467
10%	30%	500	RF (score)	0.0015	0.0038	0.0031	2.574
10%	50%	50	CCA	0.0602	0.0661	0.0323	1.097
10%	50%	50	PMM (item)	0.0265	0.0382	0.0323	1.445
10%	50%	50	PMM (score)	0.0256	0.0364	0.0323	1.425
10%	50%	50	RF (item)	0.0267	0.0333	0.0323	1.247
10%	50%	50	RF (score)	0.0275	0.0432	0.0323	1.572
10%	50%	100	CCA	0.0316	0.0316	0.0149	0.998
10%	50%	100	PMM (item)	0.0167	0.0222	0.0149	1.332
10%	50%	100	PMM (score)	0.0164	0.0213	0.0149	1.301
10%	50%	100	RF (item)	0.0157	0.0170	0.0149	1.081
10%	50%	100	RF (score)	0.0159	0.0209	0.0149	1.311
10%	50%	200	CCA	0.0150	0.0165	0.0079	1.103
10%	50%	200	PMM (item)	0.0090	0.0128	0.0079	1.418
10%	50%	200	PMM (score)	0.0088	0.0125	0.0079	1.428
10%	50%	200	RF (item)	0.0085	0.0096	0.0079	1.126
10%	50%	200	RF (score)	0.0083	0.0111	0.0079	1.334
10%	50%	300	CCA	0.0096	0.0109	0.0052	1.135
10%	50%	300	PMM (item)	0.0055	0.0088	0.0052	1.595
10%	50%	300	PMM (score)	0.0053	0.0085	0.0052	1.599
10%	50%	300	RF (item)	0.0052	0.0066	0.0052	1.266
10%	50%	300	RF (score)	0.0051	0.0073	0.0052	1.443
10%	50%	400	CCA	0.0059	0.0080	0.0038	1.356
10%	50%	400	PMM (item)	0.0036	0.0065	0.0038	1.779
10%	50%	400	PMM (score)	0.0036	0.0064	0.0038	1.764
10%	50%	400	RF (item)	0.0035	0.0050	0.0038	1.411

Table 14: Variance-related measures: main specification *(continued)*

Unit_Nonresponse	Missing_total	N	Method	Empirical_Var	Estimated_Var	True_Var	Var_Ratio
10%	50%	400	RF (score)	0.0034	0.0054	0.0038	1.566
10%	50%	500	CCA	0.0041	0.0064	0.0031	1.553
10%	50%	500	PMM (item)	0.0023	0.0052	0.0031	2.241
10%	50%	500	PMM (score)	0.0022	0.0050	0.0031	2.249
10%	50%	500	RF (item)	0.0021	0.0040	0.0031	1.877
10%	50%	500	RF (score)	0.0021	0.0043	0.0031	2.041
20%	10%	50	CCA	0.0256	0.0360	0.0323	1.405
20%	10%	50	PMM (item)	0.0222	0.0339	0.0323	1.527
20%	10%	50	PMM (score)	0.0221	0.0336	0.0323	1.520
20%	10%	50	RF (item)	0.0221	0.0328	0.0323	1.489
20%	10%	50	RF (score)	0.0221	0.0344	0.0323	1.555
20%	10%	100	CCA	0.0150	0.0167	0.0149	1.110
20%	10%	100	PMM (item)	0.0137	0.0166	0.0149	1.210
20%	10%	100	PMM (score)	0.0136	0.0165	0.0149	1.214
20%	10%	100	RF (item)	0.0137	0.0158	0.0149	1.161
20%	10%	100	RF (score)	0.0136	0.0164	0.0149	1.208
20%	10%	200	CCA	0.0069	0.0088	0.0079	1.280
20%	10%	200	PMM (item)	0.0063	0.0088	0.0079	1.401
20%	10%	200	PMM (score)	0.0062	0.0088	0.0079	1.403
20%	10%	200	RF (item)	0.0062	0.0084	0.0079	1.358
20%	10%	200	RF (score)	0.0062	0.0086	0.0079	1.391
20%	10%	300	CCA	0.0043	0.0058	0.0052	1.343
20%	10%	300	PMM (item)	0.0039	0.0059	0.0052	1.497
20%	10%	300	PMM (score)	0.0039	0.0058	0.0052	1.503
20%	10%	300	RF (item)	0.0039	0.0056	0.0052	1.432
20%	10%	300	RF (score)	0.0039	0.0057	0.0052	1.475
20%	10%	400	CCA	0.0022	0.0043	0.0038	1.923
20%	10%	400	PMM (item)	0.0020	0.0043	0.0038	2.114
20%	10%	400	PMM (score)	0.0020	0.0043	0.0038	2.105
20%	10%	400	RF (item)	0.0021	0.0042	0.0038	2.026
20%	10%	400	RF (score)	0.0021	0.0042	0.0038	2.055
20%	10%	500	CCA	0.0011	0.0034	0.0031	3.223
20%	10%	500	PMM (item)	0.0009	0.0034	0.0031	3.715
20%	10%	500	PMM (score)	0.0009	0.0034	0.0031	3.737
20%	10%	500	RF (item)	0.0009	0.0033	0.0031	3.501
20%	10%	500	RF (score)	0.0010	0.0034	0.0031	3.531
20%	20%	50	CCA	0.0306	0.0418	0.0323	1.367
20%	20%	50	PMM (item)	0.0227	0.0356	0.0323	1.571
20%	20%	50	PMM (score)	0.0226	0.0349	0.0323	1.544
20%	20%	50	RF (item)	0.0224	0.0333	0.0323	1.489
20%	20%	50	RF (score)	0.0226	0.0368	0.0323	1.626
20%	20%	100	CCA	0.0177	0.0188	0.0149	1.063
20%	20%	100	PMM (item)	0.0141	0.0178	0.0149	1.261
20%	20%	100	PMM (score)	0.0141	0.0177	0.0149	1.254
20%	20%	100	RF (item)	0.0141	0.0162	0.0149	1.155
20%	20%	100	RF (score)	0.0140	0.0174	0.0149	1.243
20%	20%	200	CCA	0.0081	0.0100	0.0079	1.230
20%	20%	200	PMM (item)	0.0069	0.0096	0.0079	1.392
20%	20%	200	PMM (score)	0.0069	0.0095	0.0079	1.390
20%	20%	200	RF (item)	0.0068	0.0088	0.0079	1.291
20%	20%	200	RF (score)	0.0067	0.0092	0.0079	1.364
20%	20%	300	CCA	0.0051	0.0066	0.0052	1.301
20%	20%	300	PMM (item)	0.0042	0.0064	0.0052	1.524
20%	20%	300	PMM (score)	0.0042	0.0064	0.0052	1.538
20%	20%	300	RF (item)	0.0042	0.0059	0.0052	1.405
20%	20%	300	RF (score)	0.0041	0.0061	0.0052	1.474
20%	20%	400	CCA	0.0028	0.0049	0.0038	1.729

Table 14: Variance-related measures: main specification *(continued)*

Unit_Nonresponse	Missing_total	N	Method	Empirical_Var	Estimated_Var	True_Var	Var_Ratio
20%	20%	400	PMM (item)	0.0024	0.0047	0.0038	2.008
20%	20%	400	PMM (score)	0.0023	0.0047	0.0038	2.027
20%	20%	400	RF (item)	0.0024	0.0044	0.0038	1.854
20%	20%	400	RF (score)	0.0023	0.0045	0.0038	1.932
20%	20%	500	CCA	0.0014	0.0039	0.0031	2.726
20%	20%	500	PMM (item)	0.0011	0.0038	0.0031	3.311
20%	20%	500	PMM (score)	0.0012	0.0037	0.0031	3.243
20%	20%	500	RF (item)	0.0012	0.0035	0.0031	3.019
20%	20%	500	RF (score)	0.0011	0.0036	0.0031	3.125
20%	30%	50	CCA	0.0377	0.0474	0.0323	1.257
20%	30%	50	PMM (item)	0.0241	0.0369	0.0323	1.533
20%	30%	50	PMM (score)	0.0236	0.0358	0.0323	1.516
20%	30%	50	RF (item)	0.0235	0.0333	0.0323	1.420
20%	30%	50	RF (score)	0.0238	0.0392	0.0323	1.649
20%	30%	100	CCA	0.0207	0.0217	0.0149	1.048
20%	30%	100	PMM (item)	0.0150	0.0191	0.0149	1.277
20%	30%	100	PMM (score)	0.0148	0.0188	0.0149	1.269
20%	30%	100	RF (item)	0.0148	0.0166	0.0149	1.120
20%	30%	100	RF (score)	0.0145	0.0184	0.0149	1.269
20%	30%	200	CCA	0.0096	0.0115	0.0079	1.198
20%	30%	200	PMM (item)	0.0073	0.0105	0.0079	1.437
20%	30%	200	PMM (score)	0.0072	0.0104	0.0079	1.451
20%	30%	200	RF (item)	0.0074	0.0091	0.0079	1.230
20%	30%	200	RF (score)	0.0072	0.0098	0.0079	1.360
20%	30%	300	CCA	0.0060	0.0076	0.0052	1.262
20%	30%	300	PMM (item)	0.0046	0.0071	0.0052	1.531
20%	30%	300	PMM (score)	0.0045	0.0070	0.0052	1.557
20%	30%	300	RF (item)	0.0044	0.0061	0.0052	1.381
20%	30%	300	RF (score)	0.0043	0.0065	0.0052	1.493
20%	30%	400	CCA	0.0035	0.0056	0.0038	1.615
20%	30%	400	PMM (item)	0.0028	0.0052	0.0038	1.894
20%	30%	400	PMM (score)	0.0027	0.0052	0.0038	1.905
20%	30%	400	RF (item)	0.0027	0.0046	0.0038	1.683
20%	30%	400	RF (score)	0.0027	0.0048	0.0038	1.783
20%	30%	500	CCA	0.0021	0.0045	0.0031	2.123
20%	30%	500	PMM (item)	0.0015	0.0042	0.0031	2.818
20%	30%	500	PMM (score)	0.0015	0.0041	0.0031	2.814
20%	30%	500	RF (item)	0.0015	0.0037	0.0031	2.495
20%	30%	500	RF (score)	0.0015	0.0038	0.0031	2.571
20%	50%	50	CCA	0.0611	0.0650	0.0323	1.063
20%	50%	50	PMM (item)	0.0267	0.0382	0.0323	1.429
20%	50%	50	PMM (score)	0.0253	0.0360	0.0323	1.421
20%	50%	50	RF (item)	0.0262	0.0329	0.0323	1.257
20%	50%	50	RF (score)	0.0266	0.0437	0.0323	1.645
20%	50%	100	CCA	0.0319	0.0313	0.0149	0.981
20%	50%	100	PMM (item)	0.0173	0.0222	0.0149	1.288
20%	50%	100	PMM (score)	0.0168	0.0212	0.0149	1.265
20%	50%	100	RF (item)	0.0163	0.0171	0.0149	1.048
20%	50%	100	RF (score)	0.0162	0.0209	0.0149	1.286
20%	50%	200	CCA	0.0153	0.0167	0.0079	1.087
20%	50%	200	PMM (item)	0.0090	0.0129	0.0079	1.435
20%	50%	200	PMM (score)	0.0087	0.0125	0.0079	1.449
20%	50%	200	RF (item)	0.0086	0.0096	0.0079	1.112
20%	50%	200	RF (score)	0.0084	0.0111	0.0079	1.323
20%	50%	300	CCA	0.0097	0.0109	0.0052	1.119
20%	50%	300	PMM (item)	0.0055	0.0088	0.0052	1.595
20%	50%	300	PMM (score)	0.0054	0.0086	0.0052	1.597

Table 14: Variance-related measures: main specification *(continued)*

Unit_Nonresponse	Missing_total	N	Method	Empirical_Var	Estimated_Var	True_Var	Var_Ratio
20%	50%	300	RF (item)	0.0053	0.0066	0.0052	1.252
20%	50%	300	RF (score)	0.0052	0.0074	0.0052	1.424
20%	50%	400	CCA	0.0061	0.0080	0.0038	1.311
20%	50%	400	PMM (item)	0.0038	0.0066	0.0038	1.718
20%	50%	400	PMM (score)	0.0037	0.0064	0.0038	1.721
20%	50%	400	RF (item)	0.0036	0.0049	0.0038	1.355
20%	50%	400	RF (score)	0.0036	0.0053	0.0038	1.499
20%	50%	500	CCA	0.0040	0.0064	0.0031	1.610
20%	50%	500	PMM (item)	0.0023	0.0052	0.0031	2.288
20%	50%	500	PMM (score)	0.0022	0.0051	0.0031	2.322
20%	50%	500	RF (item)	0.0020	0.0040	0.0031	1.950
20%	50%	500	RF (score)	0.0020	0.0043	0.0031	2.102
30%	10%	50	CCA	0.0247	0.0362	0.0323	1.463
30%	10%	50	PMM (item)	0.0217	0.0341	0.0323	1.569
30%	10%	50	PMM (score)	0.0215	0.0338	0.0323	1.568
30%	10%	50	RF (item)	0.0218	0.0329	0.0323	1.509
30%	10%	50	RF (score)	0.0217	0.0346	0.0323	1.599
30%	10%	100	CCA	0.0153	0.0167	0.0149	1.089
30%	10%	100	PMM (item)	0.0138	0.0166	0.0149	1.203
30%	10%	100	PMM (score)	0.0137	0.0165	0.0149	1.203
30%	10%	100	RF (item)	0.0137	0.0158	0.0149	1.154
30%	10%	100	RF (score)	0.0137	0.0164	0.0149	1.203
30%	10%	200	CCA	0.0069	0.0088	0.0079	1.279
30%	10%	200	PMM (item)	0.0063	0.0088	0.0079	1.391
30%	10%	200	PMM (score)	0.0063	0.0088	0.0079	1.392
30%	10%	200	RF (item)	0.0064	0.0084	0.0079	1.329
30%	10%	200	RF (score)	0.0064	0.0086	0.0079	1.355
30%	10%	300	CCA	0.0042	0.0058	0.0052	1.381
30%	10%	300	PMM (item)	0.0038	0.0059	0.0052	1.544
30%	10%	300	PMM (score)	0.0038	0.0059	0.0052	1.555
30%	10%	300	RF (item)	0.0038	0.0056	0.0052	1.469
30%	10%	300	RF (score)	0.0038	0.0057	0.0052	1.498
30%	10%	400	CCA	0.0022	0.0043	0.0038	1.939
30%	10%	400	PMM (item)	0.0020	0.0043	0.0038	2.120
30%	10%	400	PMM (score)	0.0020	0.0043	0.0038	2.118
30%	10%	400	RF (item)	0.0020	0.0042	0.0038	2.040
30%	10%	400	RF (score)	0.0020	0.0042	0.0038	2.081
30%	10%	500	CCA	0.0010	0.0034	0.0031	3.289
30%	10%	500	PMM (item)	0.0009	0.0034	0.0031	3.788
30%	10%	500	PMM (score)	0.0009	0.0034	0.0031	3.788
30%	10%	500	RF (item)	0.0009	0.0033	0.0031	3.532
30%	10%	500	RF (score)	0.0009	0.0034	0.0031	3.620
30%	20%	50	CCA	0.0293	0.0406	0.0323	1.388
30%	20%	50	PMM (item)	0.0226	0.0353	0.0323	1.565
30%	20%	50	PMM (score)	0.0223	0.0346	0.0323	1.554
30%	20%	50	RF (item)	0.0220	0.0331	0.0323	1.505
30%	20%	50	RF (score)	0.0220	0.0365	0.0323	1.661
30%	20%	100	CCA	0.0176	0.0188	0.0149	1.072
30%	20%	100	PMM (item)	0.0142	0.0179	0.0149	1.259
30%	20%	100	PMM (score)	0.0140	0.0177	0.0149	1.266
30%	20%	100	RF (item)	0.0140	0.0162	0.0149	1.157
30%	20%	100	RF (score)	0.0140	0.0174	0.0149	1.245
30%	20%	200	CCA	0.0081	0.0100	0.0079	1.234
30%	20%	200	PMM (item)	0.0068	0.0096	0.0079	1.408
30%	20%	200	PMM (score)	0.0068	0.0095	0.0079	1.408
30%	20%	200	RF (item)	0.0067	0.0088	0.0079	1.310
30%	20%	200	RF (score)	0.0067	0.0092	0.0079	1.373

Table 14: Variance-related measures: main specification *(continued)*

Unit_Nonresponse	Missing_total	N	Method	Empirical_Var	Estimated_Var	True_Var	Var_Ratio
30%	20%	300	CCA	0.0050	0.0066	0.0052	1.318
30%	20%	300	PMM (item)	0.0042	0.0064	0.0052	1.541
30%	20%	300	PMM (score)	0.0041	0.0064	0.0052	1.540
30%	20%	300	RF (item)	0.0042	0.0059	0.0052	1.420
30%	20%	300	RF (score)	0.0041	0.0061	0.0052	1.485
30%	20%	400	CCA	0.0028	0.0049	0.0038	1.743
30%	20%	400	PMM (item)	0.0024	0.0048	0.0038	2.008
30%	20%	400	PMM (score)	0.0023	0.0047	0.0038	2.015
30%	20%	400	RF (item)	0.0024	0.0044	0.0038	1.823
30%	20%	400	RF (score)	0.0024	0.0045	0.0038	1.905
30%	20%	500	CCA	0.0015	0.0039	0.0031	2.613
30%	20%	500	PMM (item)	0.0012	0.0038	0.0031	3.209
30%	20%	500	PMM (score)	0.0012	0.0037	0.0031	3.190
30%	20%	500	RF (item)	0.0012	0.0035	0.0031	2.900
30%	20%	500	RF (score)	0.0012	0.0036	0.0031	2.991
30%	30%	50	CCA	0.0364	0.0469	0.0323	1.288
30%	30%	50	PMM (item)	0.0237	0.0367	0.0323	1.547
30%	30%	50	PMM (score)	0.0233	0.0356	0.0323	1.525
30%	30%	50	RF (item)	0.0234	0.0333	0.0323	1.421
30%	30%	50	RF (score)	0.0239	0.0388	0.0323	1.625
30%	30%	100	CCA	0.0211	0.0218	0.0149	1.030
30%	30%	100	PMM (item)	0.0149	0.0194	0.0149	1.295
30%	30%	100	PMM (score)	0.0148	0.0190	0.0149	1.286
30%	30%	100	RF (item)	0.0145	0.0166	0.0149	1.148
30%	30%	100	RF (score)	0.0145	0.0186	0.0149	1.287
30%	30%	200	CCA	0.0099	0.0115	0.0079	1.171
30%	30%	200	PMM (item)	0.0076	0.0105	0.0079	1.390
30%	30%	200	PMM (score)	0.0074	0.0104	0.0079	1.399
30%	30%	200	RF (item)	0.0075	0.0090	0.0079	1.206
30%	30%	200	RF (score)	0.0073	0.0098	0.0079	1.345
30%	30%	300	CCA	0.0061	0.0076	0.0052	1.249
30%	30%	300	PMM (item)	0.0047	0.0071	0.0052	1.509
30%	30%	300	PMM (score)	0.0045	0.0070	0.0052	1.532
30%	30%	300	RF (item)	0.0044	0.0061	0.0052	1.390
30%	30%	300	RF (score)	0.0044	0.0065	0.0052	1.479
30%	30%	400	CCA	0.0035	0.0056	0.0038	1.583
30%	30%	400	PMM (item)	0.0027	0.0052	0.0038	1.924
30%	30%	400	PMM (score)	0.0027	0.0051	0.0038	1.938
30%	30%	400	RF (item)	0.0027	0.0046	0.0038	1.710
30%	30%	400	RF (score)	0.0027	0.0048	0.0038	1.814
30%	30%	500	CCA	0.0020	0.0045	0.0031	2.183
30%	30%	500	PMM (item)	0.0015	0.0042	0.0031	2.857
30%	30%	500	PMM (score)	0.0014	0.0041	0.0031	2.915
30%	30%	500	RF (item)	0.0014	0.0037	0.0031	2.586
30%	30%	500	RF (score)	0.0014	0.0038	0.0031	2.671
30%	50%	50	CCA	0.0599	0.0671	0.0323	1.120
30%	50%	50	PMM (item)	0.0277	0.0386	0.0323	1.395
30%	50%	50	PMM (score)	0.0267	0.0362	0.0323	1.355
30%	50%	50	RF (item)	0.0267	0.0326	0.0323	1.222
30%	50%	50	RF (score)	0.0273	0.0430	0.0323	1.577
30%	50%	100	CCA	0.0314	0.0318	0.0149	1.012
30%	50%	100	PMM (item)	0.0164	0.0225	0.0149	1.378
30%	50%	100	PMM (score)	0.0161	0.0219	0.0149	1.363
30%	50%	100	RF (item)	0.0154	0.0172	0.0149	1.119
30%	50%	100	RF (score)	0.0153	0.0214	0.0149	1.396
30%	50%	200	CCA	0.0155	0.0167	0.0079	1.076
30%	50%	200	PMM (item)	0.0093	0.0129	0.0079	1.393

Table 14: Variance-related measures: main specification *(continued)*

Unit_Nonresponse	Missing_total	N	Method	Empirical_Var	Estimated_Var	True_Var	Var_Ratio
30%	50%	200	PMM (score)	0.0090	0.0126	0.0079	1.400
30%	50%	200	RF (item)	0.0087	0.0096	0.0079	1.095
30%	50%	200	RF (score)	0.0085	0.0110	0.0079	1.295
30%	50%	300	CCA	0.0097	0.0109	0.0052	1.124
30%	50%	300	PMM (item)	0.0056	0.0087	0.0052	1.549
30%	50%	300	PMM (score)	0.0055	0.0086	0.0052	1.570
30%	50%	300	RF (item)	0.0053	0.0066	0.0052	1.240
30%	50%	300	RF (score)	0.0052	0.0073	0.0052	1.403
30%	50%	400	CCA	0.0059	0.0080	0.0038	1.363
30%	50%	400	PMM (item)	0.0038	0.0064	0.0038	1.705
30%	50%	400	PMM (score)	0.0037	0.0063	0.0038	1.723
30%	50%	400	RF (item)	0.0035	0.0050	0.0038	1.417
30%	50%	400	RF (score)	0.0034	0.0054	0.0038	1.579
30%	50%	500	CCA	0.0039	0.0064	0.0031	1.629
30%	50%	500	PMM (item)	0.0023	0.0051	0.0031	2.202
30%	50%	500	PMM (score)	0.0022	0.0051	0.0031	2.273
30%	50%	500	RF (item)	0.0022	0.0040	0.0031	1.814
30%	50%	500	RF (score)	0.0022	0.0043	0.0031	1.945
50%	10%	50	CCA	0.0255	0.0360	0.0323	1.411
50%	10%	50	PMM (item)	0.0220	0.0339	0.0323	1.539
50%	10%	50	PMM (score)	0.0219	0.0337	0.0323	1.539
50%	10%	50	RF (item)	0.0220	0.0329	0.0323	1.498
50%	10%	50	RF (score)	0.0220	0.0345	0.0323	1.566
50%	10%	100	CCA	0.0153	0.0167	0.0149	1.093
50%	10%	100	PMM (item)	0.0138	0.0166	0.0149	1.200
50%	10%	100	PMM (score)	0.0138	0.0165	0.0149	1.200
50%	10%	100	RF (item)	0.0136	0.0159	0.0149	1.164
50%	10%	100	RF (score)	0.0137	0.0164	0.0149	1.201
50%	10%	200	CCA	0.0070	0.0088	0.0079	1.266
50%	10%	200	PMM (item)	0.0064	0.0089	0.0079	1.384
50%	10%	200	PMM (score)	0.0064	0.0088	0.0079	1.382
50%	10%	200	RF (item)	0.0064	0.0084	0.0079	1.327
50%	10%	200	RF (score)	0.0063	0.0086	0.0079	1.363
50%	10%	300	CCA	0.0044	0.0058	0.0052	1.333
50%	10%	300	PMM (item)	0.0039	0.0059	0.0052	1.496
50%	10%	300	PMM (score)	0.0039	0.0058	0.0052	1.490
50%	10%	300	RF (item)	0.0039	0.0056	0.0052	1.434
50%	10%	300	RF (score)	0.0039	0.0057	0.0052	1.456
50%	10%	400	CCA	0.0022	0.0043	0.0038	1.923
50%	10%	400	PMM (item)	0.0021	0.0043	0.0038	2.085
50%	10%	400	PMM (score)	0.0021	0.0043	0.0038	2.082
50%	10%	400	RF (item)	0.0021	0.0042	0.0038	2.028
50%	10%	400	RF (score)	0.0021	0.0042	0.0038	2.059
50%	10%	500	CCA	0.0011	0.0034	0.0031	3.242
50%	10%	500	PMM (item)	0.0009	0.0034	0.0031	3.795
50%	10%	500	PMM (score)	0.0009	0.0034	0.0031	3.797
50%	10%	500	RF (item)	0.0009	0.0033	0.0031	3.544
50%	10%	500	RF (score)	0.0009	0.0034	0.0031	3.624
50%	20%	50	CCA	0.0304	0.0410	0.0323	1.349
50%	20%	50	PMM (item)	0.0225	0.0356	0.0323	1.582
50%	20%	50	PMM (score)	0.0223	0.0349	0.0323	1.566
50%	20%	50	RF (item)	0.0223	0.0332	0.0323	1.491
50%	20%	50	RF (score)	0.0222	0.0367	0.0323	1.651
50%	20%	100	CCA	0.0176	0.0187	0.0149	1.064
50%	20%	100	PMM (item)	0.0145	0.0178	0.0149	1.224
50%	20%	100	PMM (score)	0.0144	0.0176	0.0149	1.219
50%	20%	100	RF (item)	0.0140	0.0161	0.0149	1.148

Table 14: Variance-related measures: main specification *(continued)*

Unit_Nonresponse	Missing_total	N	Method	Empirical_Var	Estimated_Var	True_Var	Var_Ratio
50%	20%	100	RF (score)	0.0140	0.0173	0.0149	1.236
50%	20%	200	CCA	0.0080	0.0100	0.0079	1.256
50%	20%	200	PMM (item)	0.0068	0.0097	0.0079	1.427
50%	20%	200	PMM (score)	0.0067	0.0096	0.0079	1.419
50%	20%	200	RF (item)	0.0068	0.0088	0.0079	1.305
50%	20%	200	RF (score)	0.0067	0.0092	0.0079	1.381
50%	20%	300	CCA	0.0051	0.0066	0.0052	1.289
50%	20%	300	PMM (item)	0.0042	0.0065	0.0052	1.536
50%	20%	300	PMM (score)	0.0041	0.0064	0.0052	1.546
50%	20%	300	RF (item)	0.0042	0.0059	0.0052	1.415
50%	20%	300	RF (score)	0.0041	0.0061	0.0052	1.481
50%	20%	400	CCA	0.0027	0.0048	0.0038	1.777
50%	20%	400	PMM (item)	0.0024	0.0047	0.0038	1.993
50%	20%	400	PMM (score)	0.0023	0.0047	0.0038	2.010
50%	20%	400	RF (item)	0.0024	0.0044	0.0038	1.846
50%	20%	400	RF (score)	0.0023	0.0045	0.0038	1.925
50%	20%	500	CCA	0.0015	0.0039	0.0031	2.592
50%	20%	500	PMM (item)	0.0012	0.0038	0.0031	3.236
50%	20%	500	PMM (score)	0.0011	0.0037	0.0031	3.274
50%	20%	500	RF (item)	0.0012	0.0035	0.0031	2.935
50%	20%	500	RF (score)	0.0012	0.0036	0.0031	3.018
50%	30%	50	CCA	0.0356	0.0469	0.0323	1.317
50%	30%	50	PMM (item)	0.0237	0.0366	0.0323	1.545
50%	30%	50	PMM (score)	0.0232	0.0357	0.0323	1.537
50%	30%	50	RF (item)	0.0228	0.0333	0.0323	1.460
50%	30%	50	RF (score)	0.0231	0.0386	0.0323	1.671
50%	30%	100	CCA	0.0207	0.0218	0.0149	1.055
50%	30%	100	PMM (item)	0.0152	0.0193	0.0149	1.270
50%	30%	100	PMM (score)	0.0149	0.0189	0.0149	1.265
50%	30%	100	RF (item)	0.0146	0.0166	0.0149	1.139
50%	30%	100	RF (score)	0.0144	0.0186	0.0149	1.285
50%	30%	200	CCA	0.0098	0.0115	0.0079	1.169
50%	30%	200	PMM (item)	0.0075	0.0106	0.0079	1.405
50%	30%	200	PMM (score)	0.0074	0.0104	0.0079	1.414
50%	30%	200	RF (item)	0.0074	0.0091	0.0079	1.233
50%	30%	200	RF (score)	0.0072	0.0098	0.0079	1.350
50%	30%	300	CCA	0.0062	0.0076	0.0052	1.233
50%	30%	300	PMM (item)	0.0046	0.0071	0.0052	1.528
50%	30%	300	PMM (score)	0.0046	0.0070	0.0052	1.541
50%	30%	300	RF (item)	0.0046	0.0062	0.0052	1.350
50%	30%	300	RF (score)	0.0045	0.0065	0.0052	1.435
50%	30%	400	CCA	0.0035	0.0056	0.0038	1.575
50%	30%	400	PMM (item)	0.0027	0.0052	0.0038	1.899
50%	30%	400	PMM (score)	0.0027	0.0051	0.0038	1.914
50%	30%	400	RF (item)	0.0027	0.0046	0.0038	1.708
50%	30%	400	RF (score)	0.0026	0.0048	0.0038	1.805
50%	30%	500	CCA	0.0021	0.0044	0.0031	2.138
50%	30%	500	PMM (item)	0.0015	0.0041	0.0031	2.820
50%	30%	500	PMM (score)	0.0014	0.0041	0.0031	2.853
50%	30%	500	RF (item)	0.0015	0.0037	0.0031	2.470
50%	30%	500	RF (score)	0.0015	0.0038	0.0031	2.611
50%	50%	50	CCA	0.0603	0.0652	0.0324	1.081
50%	50%	50	PMM (item)	0.0262	0.0380	0.0323	1.452
50%	50%	50	PMM (score)	0.0253	0.0361	0.0323	1.428
50%	50%	50	RF (item)	0.0259	0.0327	0.0323	1.263
50%	50%	50	RF (score)	0.0265	0.0434	0.0323	1.636
50%	50%	100	CCA	0.0314	0.0318	0.0149	1.011

Table 14: Variance-related measures: main specification *(continued)*

Unit_Nonresponse	Missing_total	N	Method	Empirical_Var	Estimated_Var	True_Var	Var_Ratio
50%	50%	100	PMM (item)	0.0176	0.0224	0.0149	1.276
50%	50%	100	PMM (score)	0.0168	0.0214	0.0149	1.280
50%	50%	100	RF (item)	0.0164	0.0172	0.0149	1.046
50%	50%	100	RF (score)	0.0160	0.0210	0.0149	1.312
50%	50%	200	CCA	0.0149	0.0167	0.0079	1.121
50%	50%	200	PMM (item)	0.0090	0.0127	0.0079	1.405
50%	50%	200	PMM (score)	0.0087	0.0125	0.0079	1.431
50%	50%	200	RF (item)	0.0084	0.0096	0.0079	1.151
50%	50%	200	RF (score)	0.0082	0.0110	0.0079	1.347
50%	50%	300	CCA	0.0096	0.0109	0.0052	1.132
50%	50%	300	PMM (item)	0.0058	0.0087	0.0052	1.516
50%	50%	300	PMM (score)	0.0056	0.0086	0.0052	1.533
50%	50%	300	RF (item)	0.0054	0.0066	0.0052	1.210
50%	50%	300	RF (score)	0.0053	0.0074	0.0052	1.386
50%	50%	400	CCA	0.0063	0.0080	0.0038	1.284
50%	50%	400	PMM (item)	0.0038	0.0065	0.0038	1.721
50%	50%	400	PMM (score)	0.0037	0.0063	0.0038	1.733
50%	50%	400	RF (item)	0.0036	0.0050	0.0038	1.382
50%	50%	400	RF (score)	0.0036	0.0054	0.0038	1.503
50%	50%	500	CCA	0.0039	0.0064	0.0031	1.621
50%	50%	500	PMM (item)	0.0023	0.0052	0.0031	2.249
50%	50%	500	PMM (score)	0.0023	0.0051	0.0031	2.245
50%	50%	500	RF (item)	0.0022	0.0040	0.0031	1.854
50%	50%	500	RF (score)	0.0021	0.0043	0.0031	2.009

1.6 Sensitivity analysis: $m = 20$ imputations

1.6.1 Plots

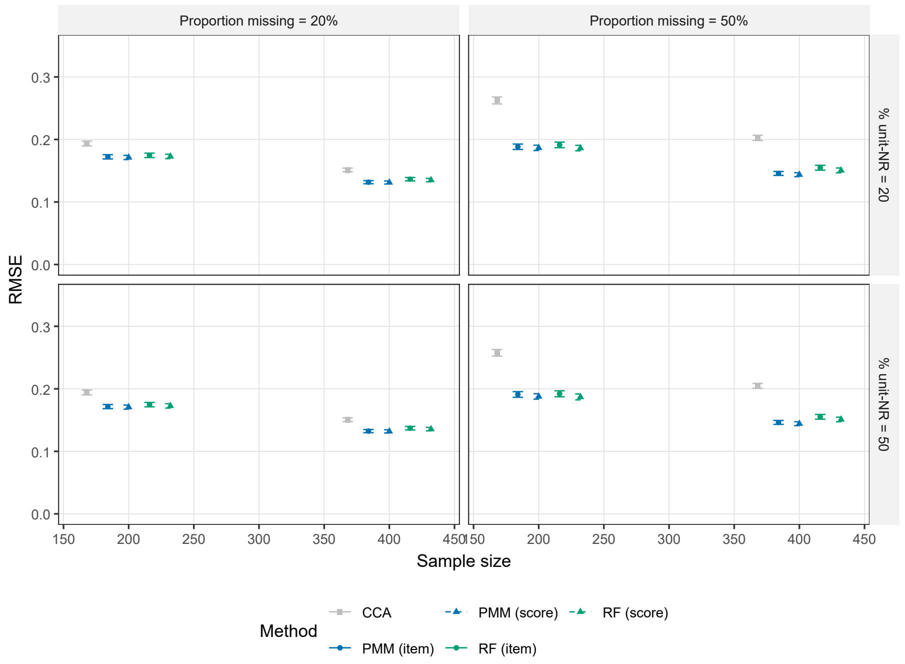

Fig. 1: RMSE estimates, $m = 20$ imputations.

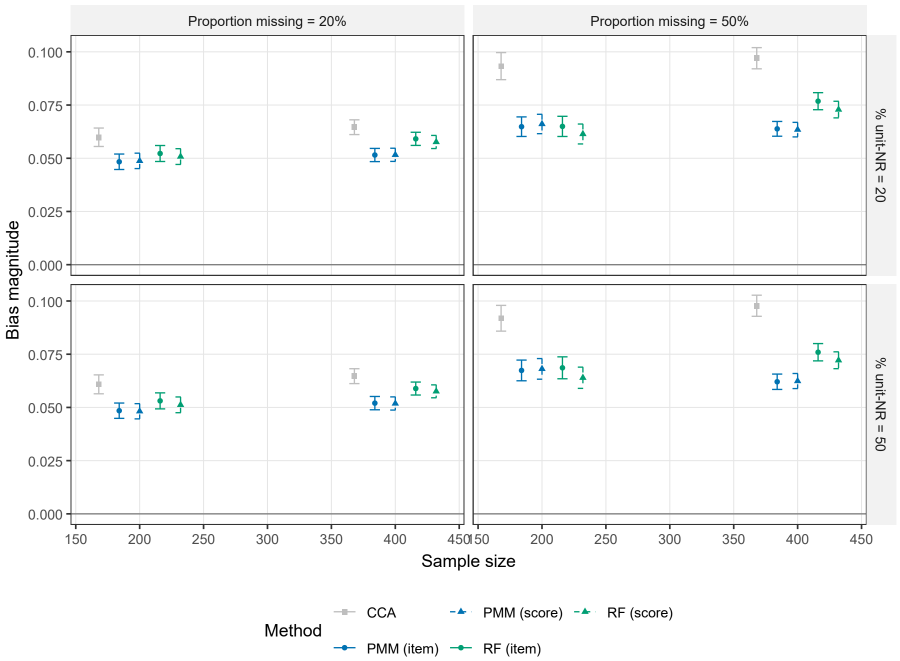

Fig. 2: Bias magnitude estimates, $m = 20$ imputations.

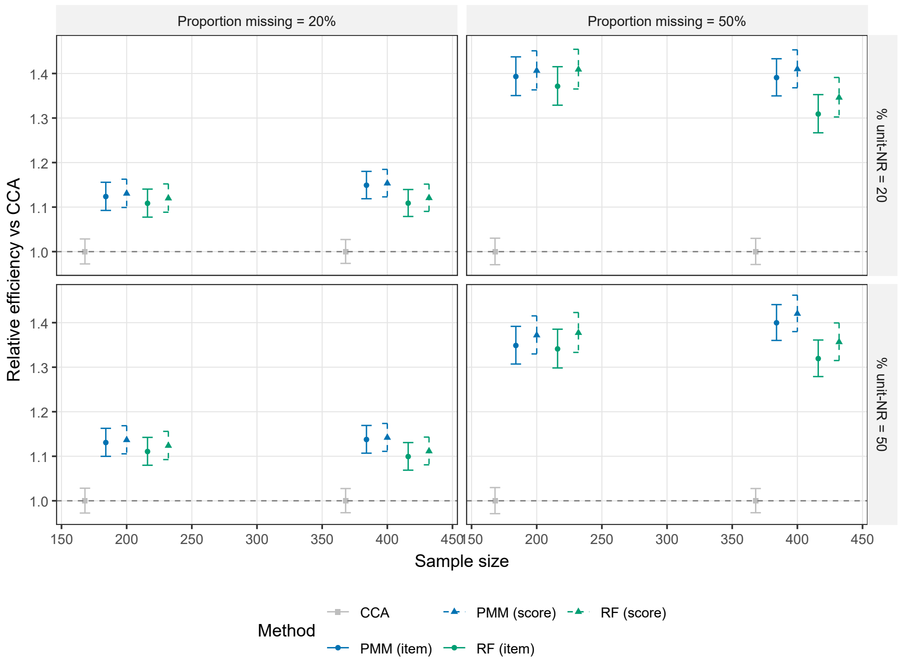

Fig. 3: Relative efficiency versus complete case analysis, $m = 20$ imputations.

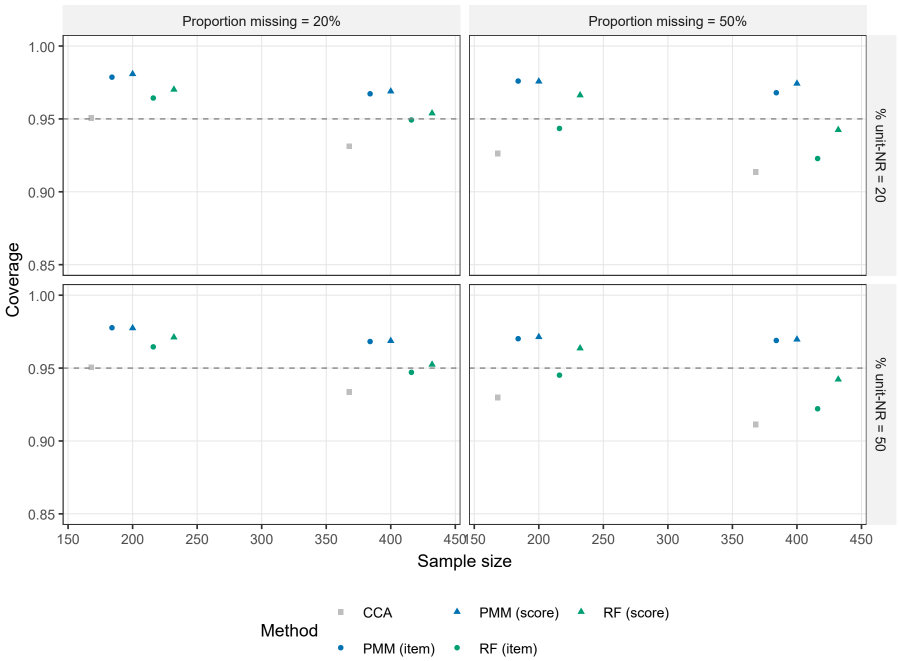

Fig. 4: Coverage estimates, $m = 20$ imputations.

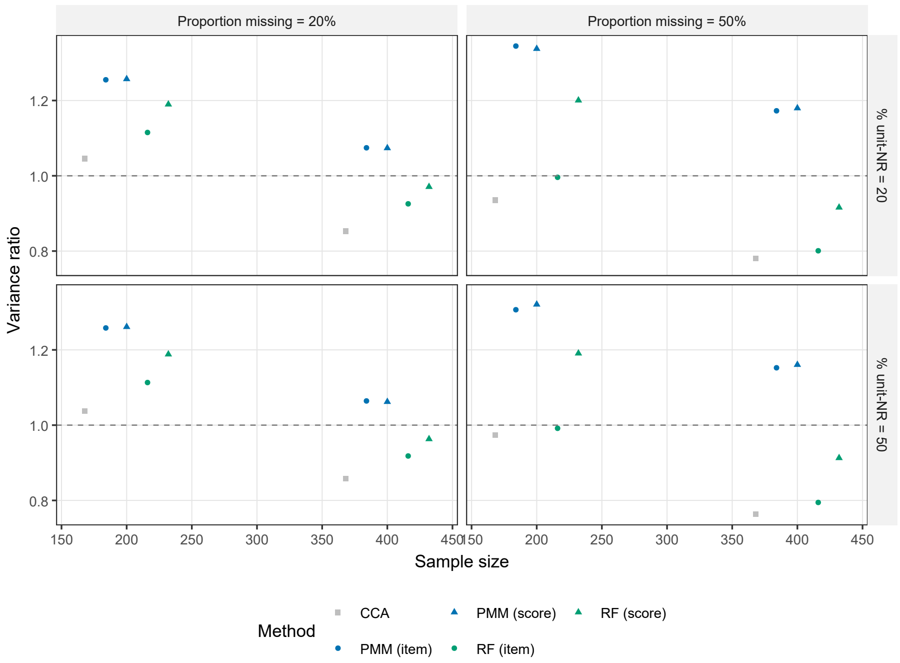

Fig. 5: Variance ratio estimates, $m = 20$ imputations.

1.6.2 Tables

Table 15: Summary performance measures: m = 20 imputations

Unit_Nonresponse	Missing_total	N	Method	Bias	RMSE	Coverage	Relative_Efficiency
20%	20%	200	CCA	0.060	0.194	0.951	1.000
20%	20%	200	PMM (item)	0.048	0.172	0.979	1.124
20%	20%	200	PMM (score)	0.049	0.171	0.981	1.130
20%	20%	200	RF (item)	0.052	0.175	0.964	1.108
20%	20%	200	RF (score)	0.051	0.173	0.970	1.120
20%	20%	400	CCA	0.065	0.151	0.931	1.000
20%	20%	400	PMM (item)	0.052	0.132	0.967	1.149
20%	20%	400	PMM (score)	0.052	0.131	0.969	1.153
20%	20%	400	RF (item)	0.059	0.136	0.949	1.109
20%	20%	400	RF (score)	0.058	0.135	0.954	1.121
20%	50%	200	CCA	0.093	0.262	0.926	1.000
20%	50%	200	PMM (item)	0.065	0.188	0.976	1.393
20%	50%	200	PMM (score)	0.066	0.187	0.976	1.406
20%	50%	200	RF (item)	0.065	0.191	0.943	1.371
20%	50%	200	RF (score)	0.061	0.186	0.966	1.409
20%	50%	400	CCA	0.097	0.203	0.914	1.000
20%	50%	400	PMM (item)	0.064	0.146	0.968	1.391
20%	50%	400	PMM (score)	0.063	0.144	0.974	1.410
20%	50%	400	RF (item)	0.077	0.155	0.923	1.309
20%	50%	400	RF (score)	0.073	0.151	0.942	1.346
50%	20%	200	CCA	0.061	0.194	0.950	1.000
50%	20%	200	PMM (item)	0.048	0.172	0.978	1.131
50%	20%	200	PMM (score)	0.048	0.171	0.977	1.137
50%	20%	200	RF (item)	0.053	0.175	0.965	1.111
50%	20%	200	RF (score)	0.051	0.173	0.971	1.124
50%	20%	400	CCA	0.065	0.151	0.934	1.000
50%	20%	400	PMM (item)	0.052	0.132	0.968	1.138
50%	20%	400	PMM (score)	0.052	0.132	0.969	1.142
50%	20%	400	RF (item)	0.059	0.137	0.947	1.099
50%	20%	400	RF (score)	0.058	0.136	0.952	1.111
50%	50%	200	CCA	0.092	0.258	0.930	1.000
50%	50%	200	PMM (item)	0.067	0.191	0.970	1.349
50%	50%	200	PMM (score)	0.068	0.188	0.971	1.372
50%	50%	200	RF (item)	0.069	0.192	0.945	1.341
50%	50%	200	RF (score)	0.064	0.187	0.964	1.377
50%	50%	400	CCA	0.098	0.205	0.911	1.000
50%	50%	400	PMM (item)	0.062	0.146	0.969	1.400
50%	50%	400	PMM (score)	0.062	0.144	0.970	1.420
50%	50%	400	RF (item)	0.076	0.155	0.922	1.319
50%	50%	400	RF (score)	0.072	0.151	0.942	1.357

Table 16: Variance-related measures: m = 20 imputations

Unit_Nonresponse	Missing_total	N	Method	Empirical_Var	Estimated_Var	True_Var	Var_Ratio
20%	20%	200	CCA	0.0093	0.0098	0.0077	1.057
20%	20%	200	PMM (item)	0.0073	0.0093	0.0077	1.273
20%	20%	200	PMM (score)	0.0072	0.0092	0.0077	1.276
20%	20%	200	RF (item)	0.0074	0.0085	0.0077	1.144
20%	20%	200	RF (score)	0.0073	0.0089	0.0077	1.217
20%	20%	400	CCA	0.0056	0.0049	0.0039	0.873
20%	20%	400	PMM (item)	0.0042	0.0047	0.0039	1.102
20%	20%	400	PMM (score)	0.0042	0.0046	0.0039	1.101
20%	20%	400	RF (item)	0.0044	0.0043	0.0039	0.982
20%	20%	400	RF (score)	0.0043	0.0044	0.0039	1.025
20%	50%	200	CCA	0.0170	0.0161	0.0077	0.945
20%	50%	200	PMM (item)	0.0086	0.0119	0.0077	1.385
20%	50%	200	PMM (score)	0.0084	0.0116	0.0077	1.389
20%	50%	200	RF (item)	0.0087	0.0091	0.0077	1.045
20%	50%	200	RF (score)	0.0083	0.0104	0.0077	1.252
20%	50%	400	CCA	0.0100	0.0080	0.0039	0.803
20%	50%	400	PMM (item)	0.0051	0.0062	0.0039	1.218
20%	50%	400	PMM (score)	0.0049	0.0061	0.0039	1.231
20%	50%	400	RF (item)	0.0052	0.0048	0.0039	0.915
20%	50%	400	RF (score)	0.0050	0.0052	0.0039	1.030
50%	20%	200	CCA	0.0093	0.0098	0.0077	1.047
50%	20%	200	PMM (item)	0.0073	0.0093	0.0077	1.276
50%	20%	200	PMM (score)	0.0072	0.0092	0.0077	1.279
50%	20%	200	RF (item)	0.0074	0.0085	0.0077	1.142
50%	20%	200	RF (score)	0.0073	0.0089	0.0077	1.214
50%	20%	400	CCA	0.0055	0.0049	0.0039	0.878
50%	20%	400	PMM (item)	0.0043	0.0047	0.0039	1.087
50%	20%	400	PMM (score)	0.0043	0.0046	0.0039	1.086
50%	20%	400	RF (item)	0.0044	0.0043	0.0039	0.971
50%	20%	400	RF (score)	0.0044	0.0044	0.0039	1.014
50%	50%	200	CCA	0.0164	0.0162	0.0077	0.986
50%	50%	200	PMM (item)	0.0088	0.0119	0.0077	1.360
50%	50%	200	PMM (score)	0.0084	0.0116	0.0077	1.385
50%	50%	200	RF (item)	0.0087	0.0091	0.0077	1.057
50%	50%	200	RF (score)	0.0083	0.0104	0.0077	1.255
50%	50%	400	CCA	0.0101	0.0080	0.0039	0.790
50%	50%	400	PMM (item)	0.0051	0.0062	0.0039	1.198
50%	50%	400	PMM (score)	0.0050	0.0060	0.0039	1.214
50%	50%	400	RF (item)	0.0052	0.0048	0.0039	0.913
50%	50%	400	RF (score)	0.0050	0.0052	0.0039	1.034

1.7 Sensitivity analysis: "don't know" assigned a value of 2.5

1.7.1 Plots

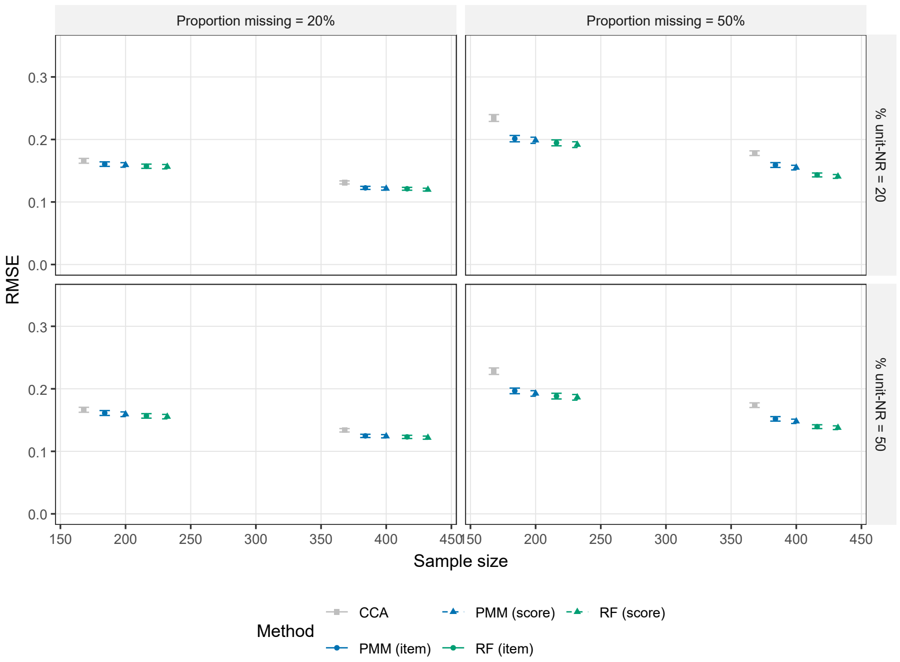

Fig. 6: RMSE estimates, "don't know" assigned a value of 2.5.

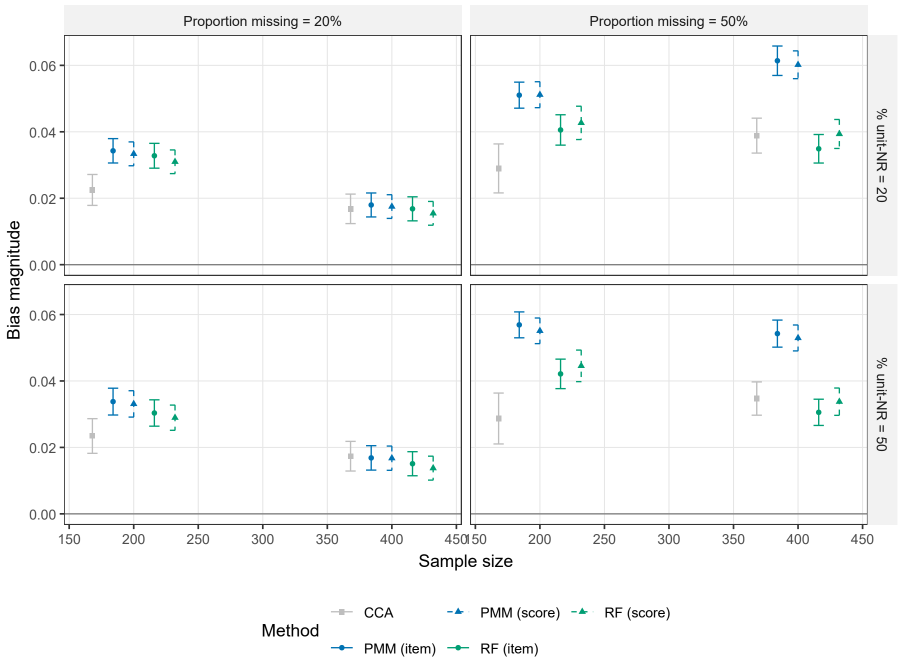

Fig. 7: Bias magnitude estimates, "don't know" assigned a value of 2.5.

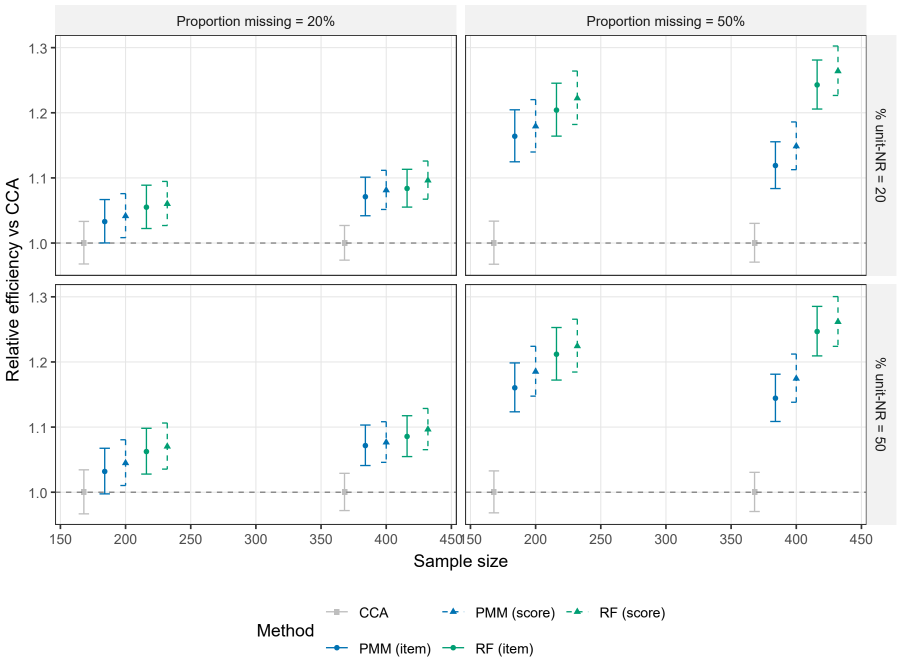

Fig. 8: Relative efficiency versus complete case analysis, "don't know" assigned a value of 2.5.

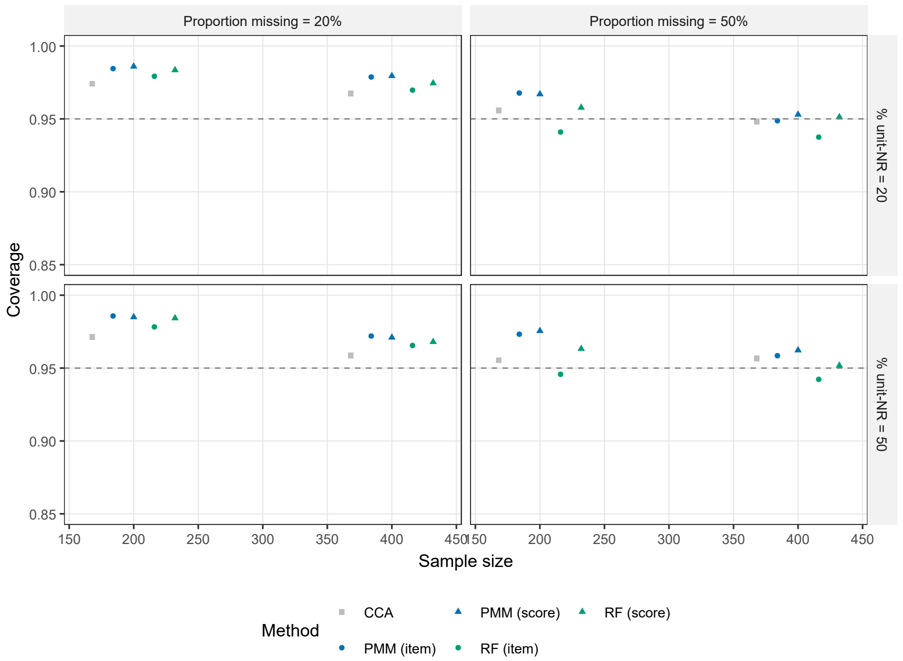

Fig. 9: Coverage estimates, "don't know" assigned a value of 2.5.

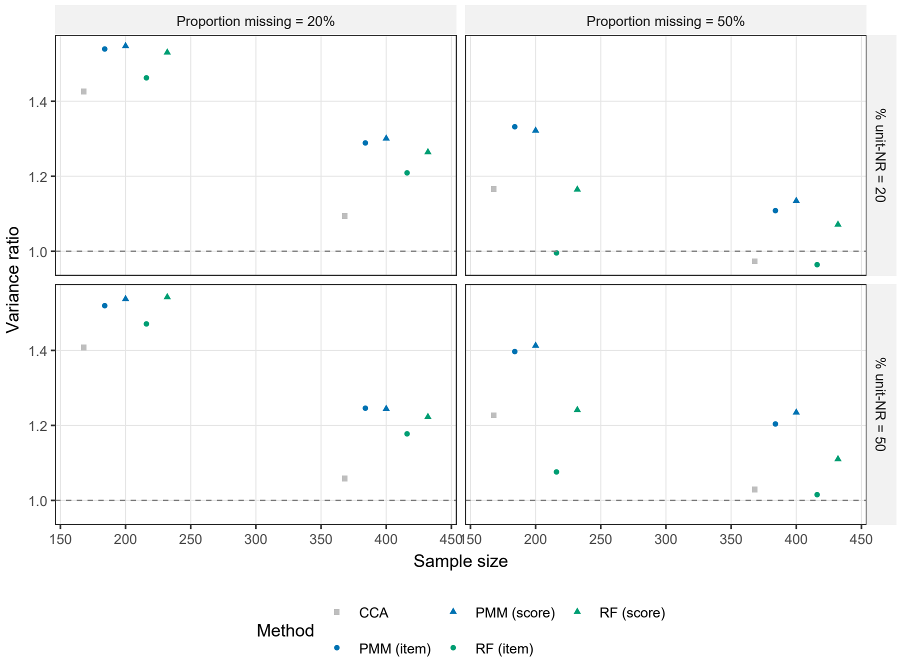

Fig. 10: Variance ratio estimates, “don’t know” assigned a value of 2.5.

1.7.2 Tables

Table 17: Summary performance measures: "don't know" assigned a value of 2.5

Unit_Nonresponse	Missing_total	N	Method	Bias	RMSE	Coverage	Relative_Efficiency
20%	20%	200	CCA	0.022	0.166	0.974	1.000
20%	20%	200	PMM (item)	0.034	0.161	0.985	1.033
20%	20%	200	PMM (score)	0.033	0.159	0.986	1.042
20%	20%	200	RF (item)	0.033	0.157	0.979	1.055
20%	20%	200	RF (score)	0.031	0.156	0.984	1.060
20%	20%	400	CCA	0.017	0.131	0.968	1.000
20%	20%	400	PMM (item)	0.018	0.123	0.979	1.071
20%	20%	400	PMM (score)	0.017	0.121	0.980	1.081
20%	20%	400	RF (item)	0.017	0.121	0.970	1.084
20%	20%	400	RF (score)	0.015	0.120	0.975	1.096
20%	50%	200	CCA	0.029	0.234	0.956	1.000
20%	50%	200	PMM (item)	0.051	0.201	0.968	1.164
20%	50%	200	PMM (score)	0.051	0.199	0.967	1.179
20%	50%	200	RF (item)	0.041	0.195	0.941	1.204
20%	50%	200	RF (score)	0.043	0.192	0.958	1.222
20%	50%	400	CCA	0.039	0.178	0.948	1.000
20%	50%	400	PMM (item)	0.061	0.159	0.949	1.119
20%	50%	400	PMM (score)	0.060	0.155	0.953	1.149
20%	50%	400	RF (item)	0.035	0.143	0.938	1.243
20%	50%	400	RF (score)	0.039	0.141	0.951	1.264
50%	20%	200	CCA	0.023	0.166	0.971	1.000
50%	20%	200	PMM (item)	0.034	0.161	0.986	1.032
50%	20%	200	PMM (score)	0.033	0.159	0.985	1.045
50%	20%	200	RF (item)	0.030	0.157	0.978	1.062
50%	20%	200	RF (score)	0.029	0.156	0.984	1.070
50%	20%	400	CCA	0.017	0.134	0.959	1.000
50%	20%	400	PMM (item)	0.017	0.125	0.972	1.072
50%	20%	400	PMM (score)	0.017	0.124	0.971	1.077
50%	20%	400	RF (item)	0.015	0.123	0.966	1.086
50%	20%	400	RF (score)	0.014	0.122	0.968	1.096
50%	50%	200	CCA	0.029	0.228	0.956	1.000
50%	50%	200	PMM (item)	0.057	0.197	0.973	1.160
50%	50%	200	PMM (score)	0.055	0.193	0.976	1.185
50%	50%	200	RF (item)	0.042	0.188	0.946	1.212
50%	50%	200	RF (score)	0.045	0.186	0.963	1.224
50%	50%	400	CCA	0.035	0.174	0.957	1.000
50%	50%	400	PMM (item)	0.054	0.152	0.959	1.144
50%	50%	400	PMM (score)	0.053	0.148	0.962	1.175
50%	50%	400	RF (item)	0.031	0.139	0.942	1.247
50%	50%	400	RF (score)	0.034	0.138	0.952	1.262

Table 18: Variance-related measures: ”don’t know” assigned a value of 2.5

Unit_Nonresponse	Missing_total	N	Method	Empirical_Var	Estimated_Var	True_Var	Var_Ratio
20%	20%	200	CCA	0.0069	0.0098	0.0079	1.429
20%	20%	200	PMM (item)	0.0064	0.0099	0.0079	1.551
20%	20%	200	PMM (score)	0.0063	0.0098	0.0079	1.559
20%	20%	200	RF (item)	0.0061	0.0090	0.0079	1.483
20%	20%	200	RF (score)	0.0061	0.0094	0.0079	1.544
20%	20%	400	CCA	0.0043	0.0047	0.0038	1.093
20%	20%	400	PMM (item)	0.0038	0.0048	0.0038	1.289
20%	20%	400	PMM (score)	0.0037	0.0048	0.0038	1.301
20%	20%	400	RF (item)	0.0036	0.0044	0.0038	1.222
20%	20%	400	RF (score)	0.0036	0.0045	0.0038	1.271
20%	50%	200	CCA	0.0136	0.0160	0.0079	1.174
20%	50%	200	PMM (item)	0.0101	0.0135	0.0079	1.332
20%	50%	200	PMM (score)	0.0099	0.0130	0.0079	1.322
20%	50%	200	RF (item)	0.0095	0.0094	0.0079	0.997
20%	50%	200	RF (score)	0.0091	0.0107	0.0079	1.171
20%	50%	400	CCA	0.0078	0.0077	0.0038	0.994
20%	50%	400	PMM (item)	0.0063	0.0070	0.0038	1.115
20%	50%	400	PMM (score)	0.0060	0.0068	0.0038	1.142
20%	50%	400	RF (item)	0.0051	0.0049	0.0038	0.964
20%	50%	400	RF (score)	0.0049	0.0053	0.0038	1.078
50%	20%	200	CCA	0.0069	0.0098	0.0079	1.409
50%	20%	200	PMM (item)	0.0065	0.0099	0.0079	1.526
50%	20%	200	PMM (score)	0.0063	0.0098	0.0079	1.545
50%	20%	200	RF (item)	0.0061	0.0090	0.0079	1.484
50%	20%	200	RF (score)	0.0060	0.0093	0.0079	1.551
50%	20%	400	CCA	0.0045	0.0047	0.0038	1.058
50%	20%	400	PMM (item)	0.0039	0.0048	0.0038	1.246
50%	20%	400	PMM (score)	0.0039	0.0048	0.0038	1.244
50%	20%	400	RF (item)	0.0038	0.0045	0.0038	1.186
50%	20%	400	RF (score)	0.0037	0.0045	0.0038	1.226
50%	50%	200	CCA	0.0130	0.0160	0.0079	1.232
50%	50%	200	PMM (item)	0.0097	0.0135	0.0079	1.399
50%	50%	200	PMM (score)	0.0093	0.0131	0.0079	1.414
50%	50%	200	RF (item)	0.0089	0.0095	0.0079	1.076
50%	50%	200	RF (score)	0.0087	0.0108	0.0079	1.244
50%	50%	400	CCA	0.0074	0.0078	0.0038	1.044
50%	50%	400	PMM (item)	0.0058	0.0069	0.0038	1.207
50%	50%	400	PMM (score)	0.0055	0.0068	0.0038	1.238
50%	50%	400	RF (item)	0.0049	0.0049	0.0038	1.015
50%	50%	400	RF (score)	0.0047	0.0053	0.0038	1.114
